# Safety and feasibility of anti-CD19 CAR T cells expressing inducible IL-7 and CCL19 in patients with relapsed or refractory large B-cell lymphoma

**DOI:** 10.1038/s41421-023-00625-0

**Published:** 2024-01-09

**Authors:** Wen Lei, Ai Zhao, Hui Liu, Chunmei Yang, Cheng Wei, Shanshan Guo, Zhilu Chen, Qunyi Guo, Linjie Li, Mingzhe Zhao, Gongqiang Wu, Guifang Ouyang, Ming Liu, Jinyi Zhang, Jimin Gao, Wenbin Qian

**Affiliations:** 1 Department of Hematology, The Second Affiliated Hospital, College of Medicine, Zhejiang University, Hangzhou, Zhejiang China; 2 Institute of Hematology, the First Affiliated Hospital, College of Medicine, Zhejiang University, Hangzhou, Zhejiang China; 3 Key Laboratory of Laboratory Medicine, Ministry of Education, School of Laboratory Medicine and Life Sciences, Wenzhou Medical University, Wenzhou, Zhejiang China; 4 Department of Geriatrics, Affiliated Hangzhou First People’s Hospital, Westlake University School of Medicine, Hangzhou, Zhejiang China; 5 Department of Hematology, Tongde Hospital of Zhejiang Province, Hangzhou, Zhejiang China; 6 Department of Hematology, Taizhou Hospital of Zhejiang Province, Wenzhou Medical University, Linhai, Zhejiang China; 7 Department of Hematology, Lishui Municipal Central Hospital, Lishui, Zhejiang China; 8 Department of Hematology, Jinhua Municipal Central Hospital, Jinhua, Zhejiang China; 9 Department of Hematology, Dongyang People’s Hospital, Wenzhou Medical University, Dongyang, Zhejiang China; 10 Ningbo Clinical Research Center for Hematological Tumor Diseases, Department of hematology, the First Affiliated Hospital of Ningbo University, Ningbo, Zhejiang China; 11 Oujiang Laboratory, Zhejiang Lab for Regenerative Medicine, Vision and Brain Health, Eye Hospital, Wenzhou Medical University, Wenzhou, Zhejiang China; 12 Hangzhou Qilan Biomedical Technology Co., Ltd, Hangzhou, Zhejiang China

**Keywords:** B-cell lymphoma, Immunoediting

## Abstract

Although CD19-specific chimeric antigen receptor (CAR) T cells are curative for patients with relapsed or refractory large B-cell lymphoma (R/R LBCL), disease relapse with tumor antigen-positive remains a challenge. Cytokine/chemokine-expressing CAR-T cells could overcome a suppressive milieu, but the clinical safety and efficacy of this CAR-T therapy remain unclear. Here we report the preclinical development of CD19-specific CAR-T cells capable of expressing interleukin (IL)-7 and chemokine (C-C motif) ligand (CCL)-19 upon CD19 engagement (referred to as 7 × 19 CAR-T cells) and results from a phase 1 and expansion phase trial of 7 × 19 CAR-T cell therapy in patients with R/R LBCL (NCT03258047). In dose-escalation phase, there were no dose-limiting toxicities observed. 39 patients with R/R LBCL received 7 × 19 CAR-T with doses ranged from 0.5 × 10^6^–4.0 × 10^6^ cells per kg body weight. Grade 3 cytokine release syndrome occurred in 5 (12.8%) patients and ≥ grade 3 neurotoxicity in 4 (10.3%) patients. The overall response rate at 3 months post-single infusion was 79.5% (complete remission, 56.4%; partial response, 23.1%). With a median follow-up of 32 months, the median progression-free survival was 13 months, and median overall survival was not reached, with an estimated rate of 53.8% (95% CI, 40.3% to 72.0%) at two years. Together, these long-term follow-up data from the multicenter clinical study suggest that 7 × 19 CAR-T cells can induce durable responses with a median overall survival of greater than 2 years, and have a manageable safety profile in patients with R/R LBCL.

## Introduction

Anti-CD19 chimeric antigen receptor (CAR) T cells have the potential to be a curative therapy for lymphoma patients. However, most patients either fail to respond to this treatment or relapse after achieving initial remission^1–3^. To improve the therapeutic benefits of CAR-T cells, many innovative CAR designs have been developed to enhance the antitumor efficiency and overcome treatment resistance related to inadequate expansion, infiltration, and persistence of CAR-T cells^4–7^. In this regard, fourth-generation CARs (referred to as “armored” CARs) that incorporate cytokines (IL-7, IL-15, or IL-21) are being developed to improve CAR-T cell persistence, tumor targeting, and antitumor capacity^8–12^. Another strategy is to modify CAR-T cells with the expression of a chemokine receptor or chemokine (CCL19, CCL21) that guides the CAR-T cells to the tumor site^4,13^. The feasibility of these strategies has been demonstrated in preclinical settings and evaluated in clinical trials across a range of malignancies^14–16^. Recently, anti-CD20 CAR-T cells expressing IL-7 and CCL19 have demonstrated enhanced antitumor activity compared to parental CAR-T cells and achieved complete elimination of pre-established solid tumors in mice^15,16^. Although preclinical evidence in different solid tumor models supports the potential antitumor efficacy of armored CAR-T strategies, clinical results in humans are lacking. In this study, we developed a novel CAR-T therapy for patients with R/R LBCL by engineering CD19-specific CAR-T cells to secrete IL-7 and CCL19 (7 × 19 CAR-T cells) upon CD19 antigen engagement. Preclinical study to characterize the anti-lymphoma activity of 7 × 19 CAR-T confirmed their superior tumor-targeting and anti-tumor cytotoxicity over conventional anti-CD19 CAR-T cells. Based on the results of preclinical data, we performed a multicenter phase 1 and expansion phase trial of 7 × 19 CAR T cells in adult patients with R/R LBCL (NCT03258047). Here we report the safety and efficacy of 7 × 19 CAR-T cell therapy.

## Results

### Enhanced anti-tumor capacity of 7 × 19 CAR T cells over conventional anti-CD19 CAR T cells

**Fig. 1:**
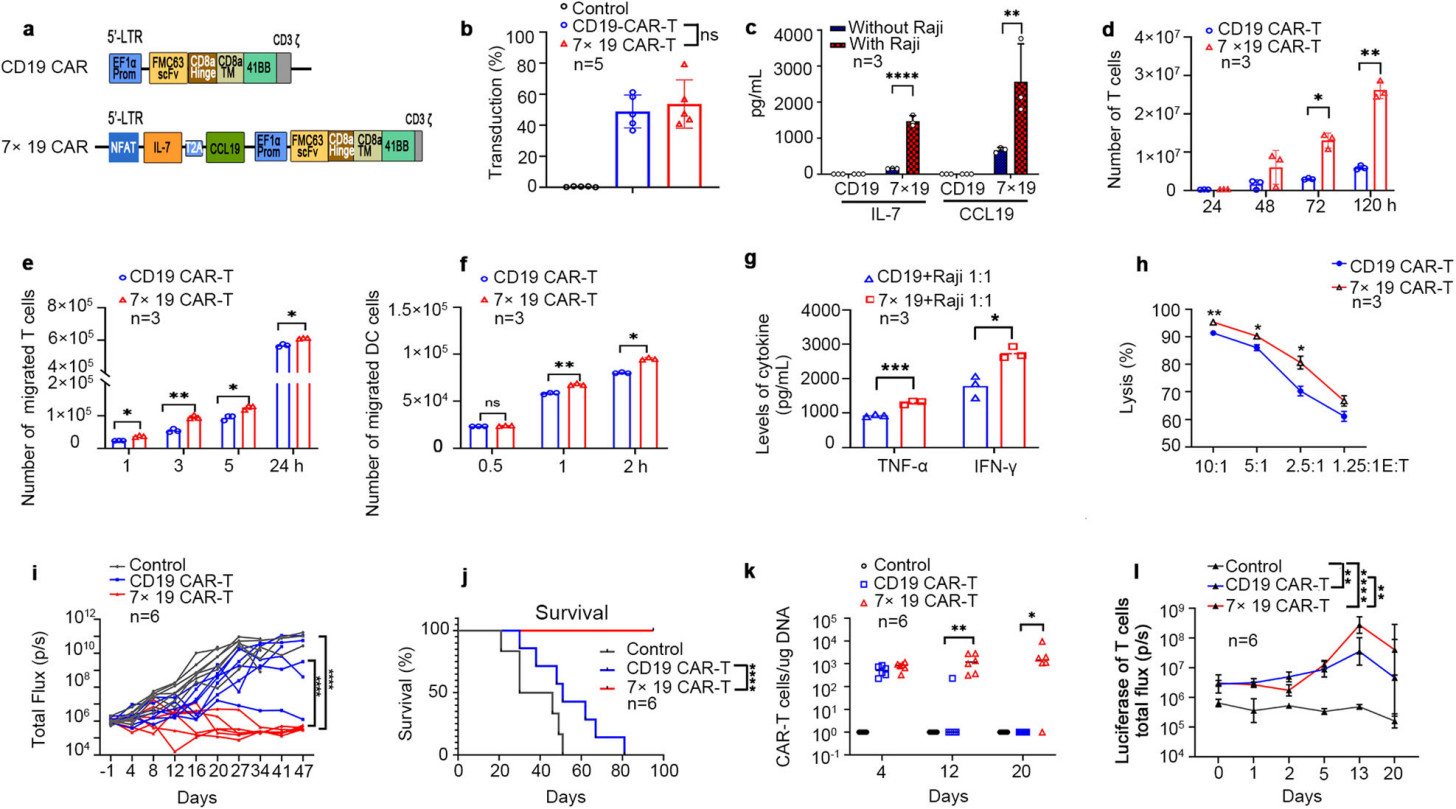
Preclinical validation of 7 × 19 CAR T cells. **a** Schematic diagram of 7 × 19 CAR construct. **b** The expression of CAR proteins in human T cells transfected with lentiviral vectors encoding anti-CD19 CAR or 7 × 19 CAR was assessed by flow cytometry after staining with anti-CD3 and anti-FMC63 scfv monoclonal antibody, respectively. **c** The concentrations of IL-7 and CCL19 in the supernatants of CD19 CAR T cells or 7 × 19 CAR T cells stimulated with or without mitomycin C-treated Raji cells for 5 days were determined by ELISA. **d** CAR T cells (1 × 10^5^) were labeled with 1 μM CSFE dye and then co-cultured with mitomycin C-treated Raji cells at a 1:1 ratio for 24 h, 48 h, 72 h and 120 h, respectively, and the proliferation of CAR T cells was assessed by CFSE dilution gated in CD3^+^ T cells. CFSE-labeled human T cells (**e**) and DCs (**f**) were cocultured with the supernatants of CAR T cells in a 24-well transwell system and the migration of T and DCs in lower chamber was determined by flow cytometry. **g** The production of TNF-α and IFN-γ in anti-CD19 CAR T or 7 × 19 CAR T cells stimulated with Raji cells at an E/T ratio of 1:1 for 24 h was determined by flow cytometry using Cytometric Bead Array (CBA) Human Th1/Th2/Th17 cytokine kit. **h** The cytotoxicity of CAR T cells against CD19-positive K562 cells was measured by luciferase-based killing assay. **i** NSG mice were treated with a single dose of CAR T cells (5 × 10^6^/per mice) or control T cells on day 7 post intravenous inoculation of Nalm-6 tumor cells. Tumor burden was monitored by bioluminescence on IVIS imaging system at the indicated days. **j** Kaplan-Meyer curve showing the survival of mice treated with anti-CD19 CAR T or 7 × 19 CAR T cells. **k** CAR transgene copy numbers in the blood of mice after CAR T cell infusion were assessed by qPCR. Logarithmic scale was used for *y*-axis. **l** After tumor inoculation in the left and right flanks, NSG mice were injected intravenously with GFP-luciferase-expressing CAR T cells and control GFP-luc-expressing T cells. CAR T cell proliferation (total flux) in vivo quantified by photons/s in mice at the indicated days. All data represent three or more independent experiments and are shown as mean ± SD. One-way analysis of variance ANOVA (**b**), two-way analysis of variance ANOVA (**c**–**f**, **h**, **k**), two-tailed Student’s *t*-test (**g**), log-rank test (**j**), and linear regression analysis (**i** and **l**). *P < 0.05, **P < 0.01, ***P < 0.001, ****P < 0.0001. ns not significant.

We developed a fourth generation anti-CD19 CAR T cells which co-expressed a CD19-specific CAR and IL-7 and CCL19 (7 × 19 CAR T cells). This 7 × 19 CAR construct was composed of DNA fragment encoding a CD19-specific CAR under the control of EF1-α promoter and DNA fragments encoding IL-7 and CCL19 separated by a self-cleaving 2 A peptide sequence under the control of 5 nuclear factor of activated T-cell (NFAT) response elements and the minimal IL-2 promoter to induce an NFAT-dependent expression of IL-7 and CCL19^17^ (Fig. 1a; Supplementary Fig. S1a). The anti-CD19 CAR- and IL-7/CCL19-containing cassettes were assembled into the backbone of a lentiviral vector and resultant 7 × 19 CAR construct transfected autogenous T cells with similar efficiency compared to anti-CD19 CAR construct (Fig. 1b). Phenotypical analyses showed that compared to anti-CD19 CAR-T, 7 × 19 CAR-T cells had significantly higher proportions of stem memory T cell (T_SCM_, CD45RA^+^CD45RO^–^CCR7^+^CD95^+^) and central memory T cell (Tcm, CD45RO^+^CD27^+^) subsets (Supplementary Fig. S1b), which are usually associated with better clinical response^18,19^. Consistently, 7 × 19 CAR-T cells expressed lower levels of the immune checkpoint molecules programmed death-1 (PD-1) or lymphocyte-activation gene 3 (LAG3) and pro-apoptotic protein Bim, but higher levels of anti-apoptotic proteins Bcl-2 and survivin and were more resistant to activation-induced apoptosis (Supplementary Fig. S1c–f).

The 7 × 19 CAR-T secreted higher levels of IL-7 and CCL19 than CD19 CAR-T cells and exhibited dramatically increased proliferation upon Raji cell stimulation (Fig. 1c, d). The medium from Raji-stimulated 7 × 19 CAR-T induced significantly enhanced migration of T cells and dendritic cells (DCs) compared to the medium of anti-CD19 CAR-T cells (Fig. 1e, f). These effects could be reversed by anti-CD127 and anti-CCR7 antibodies which blocked the IL-7 receptor and CCL19 receptor, respectively (Supplementary Fig. S1g, h), indicating that IL-7 and CCL19 produced by CAR-T cells promoted the expansion of CAR-T and chemotaxis of immune cells.

Moreover, 7 × 19 CAR-T cells exhibited enhanced in vitro cytotoxicity against CD19^+^ tumor cells relative to anti-CD19 CAR T cells (Fig. 1g, h; Supplementary Fig. S1i). We further investigated the anti-lymphoma activity of 7 × 19 CAR T cells in vivo by a single dose (5.0 × 10^6^) of intravenous (I.V.) injection of anti-CD19 CAR T, 7 × 19 CAR T or control T cells into Nalm6 xenograft NOD/SCID mice. The Nalm6-bearing mice that received anti-CD19 CAR-T cells showed an initial tumor burden decline, but eventually experienced disease progression. In contrast, administration of 7 × 19 CAR-T effectively obliterated lymphoma growth, with 5 out of 6 tumor-bearing mice achieving long-term tumor-free survival up to 95 days post-treatment (Fig. 1i, j; Supplementary Fig. S2a). Consistent with their superior anti-lymphoma activity, 7 × 19 CAR-T exhibited enhanced in vivo expansion, longer duration of persistence, and significantly increased recruitment of 7 × 19 CAR-T into tumor sites than anti-CD19 CAR-T cells (Fig. 1k, l; Supplementary Fig. S2b). These data provided the rationale for the development of clinical trials for lymphoma patients.

### Dose-escalation and expansion phase trial of 7 × 19 CAR-T cells

**Fig. 2:**
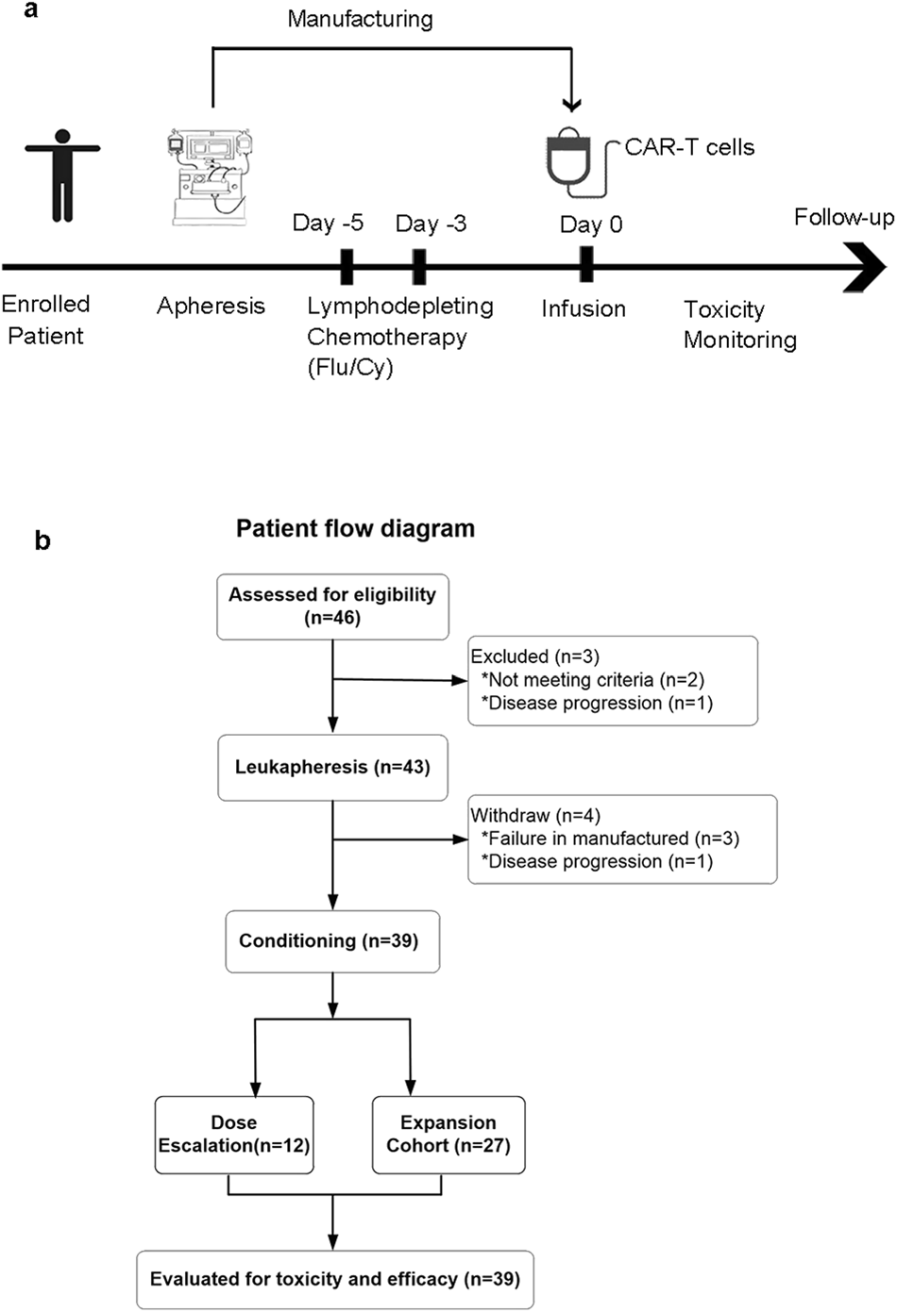
Clinical trial scheme and demographic and clinical characteristics of the enrolled patients. **a** The treatment scheme showing the enrolled patients who underwent leukapheresis and then received lympho-depleting chemotherapy. After CAR-T products were successfully manufactured, patients were treated with CAR-T cells. The efficacy and toxicity were monitored during follow-up. **b** Flow diagram describing numbers of patients enrolled in this clinical trial. Enrollment occurred to obtain peripheral blood for manufacture of the CAR-T products and then the prospective patients were required to meet eligibility to infuse CAR-T cells.

The study design and eligibility criteria are outlined in Fig. 2a and Supplementary protocol. The primary endpoint of dose-escalation phase was safety. Secondary endpoints included the rates of complete remission (CR) or partial remission (PR), as well as the frequency and severity of adverse events (AEs), expansion and persistence of 7 × 19 CAR-T cells, duration of B cell aplasia, and overall survival (OS) and progression-free survival (PFS).

Thirty-nine patients were enrolled between September 3, 2018 and November 30, 2021 in multiple centers (Fig. 2b). Of the patients who received a single infusion of 7 × 19 CAR-T cells, the median age was 55 years (range 29–73). The enrolled patients included large B-cell lymphoma (DLBCL; 33 cases), primary mediastinal B-cell lymphoma (PMBCL; 1 case), transformed follicular lymphoma (tFL; 2 cases), and mantle cell lymphoma (MCL; 3 cases). Most of the patients (66.7%) had stage III or IV disease, 64.1% had extranodal involvement, and 41% had an Eastern Cooperative Oncology Group (ECOG) performance status (PS) of 2 to 3. The median number of prior lines of therapy was 3 (range 1–6), with 51.3% of patients having refractory disease (Table 1). Among them, four patients had a history of primary refractory disease.

**Table 1:** Baseline demographic and clinical characteristics of the 39 enrolled patients.

Characteristics	No. of Patients (%) *n* = 39
**Age (years)**	
Median (range)	55 (29-73)
<60	21 (53.8%)
≥60	18 (46.2%)
**Sex**	
Male	25 (64.1%)
Female	14 (35.9%)
**History**	
DLBCL-nos	31 (79.4%)
DLBCL-Unclassified	1 (2.6%)
HGBL-DHL	1 (2.6%)
MCL	3 (7.4%)
PMLBCL	1 (2.6%)
tFL	2 (5.1%)
**Disease stage at study entry**	
I or II	13 (33.3%)
III or IV	26 (66.7%)
**IPI score**	
0-1	6 (15.4%)
2-3	21 (53.8%)
4-5	12 (30.8%)
**ECOG score**	
0-1	23 (58.9%)
2-3	16 (41.0%)
**Extranodal organ involvement**	
Yes	25 (64.1%)
No	14 (35.9%)
**No.of previous lines of antineoplastic therapy**	
Median (range)	2 (1-6)
1	4 (10.2)
2	15 (38.5)
3-6	20 (51.3%)
**No. of prior treatment regimen**	
Median (range)	9 (4-43)
<10	21 (53.8%)
≥10	18 (46.2%)
**Prior target therapy or ASCT**	
BTK inhibitor	5 (12.8%)
ASCT	3 (7.7%)
Neither BTK inhibitor nor ASCT	31 (79.5%)
**Refractory or Relapse**	
Refractory	19 (51.3%)
Relapse	20 (48.7%)
**Bulky disease** (%)^a^	
Yes	11 (28.20%
No	28 (71.79%)

DLBCL-nos diffuse large B-cell lymphoma not otherwise specified; MCL mantle cell lymphoma; tFL transformed follicular lymphoma; PMLBCL primary mediastinal large B cell lymphoma; ECOG Eastern Cooperative Oncology Group; IPI International Prognostic Index; ASCT autologous stem cell transplantation; BTK Bruton’s tyrosine kinase.

^a^Bulky disease was defined as the presence of any mass with a single diameter > 10 cm.

### Mild toxicity of 7 × 19 CAR-T cells

No patient developed dose-limiting toxicity (DLT), and the maximum tolerated dose (MTD) was not reached in dose-escalation phase. Therefore, the data of all 39 cases were combined for analysis. The most common AEs in the first 4 weeks after CAR-T cell infusion were granulocytopenia (94.9%), anemia (87.2%), and fever (74.4%), with ≥ grade 3 granulocytopenia (76.9%) and anemia (41.0%) (Table 2), which was similar to that in anti-CD19 CAR-T therapies (78% of neutropenia, 43% of anemia and 38% thrombocytopenia)^3,20^. 12 (34.3%) of the assessable 35 patients showed neutropenia at 3 months after CAR-T infusion. Continued B-cell aplasia, a phenotype that often predicts good response to anti-CD19 CAR-T therapy^3,21^, was observed in 18 of 25 patients at 9 months, while 13 of 24 cases remained in B-cell aplasia at 12 months (Supplementary Fig. S3).

**Table 2:** Adverse events among all 39 treated patients.

Adverse events	Any	Grade 1	Grade 2	Grade 3	Grade 4
CRS	29 (74.4%)	9 (23.1%)	15 (38.5%)	5 (12.8%)	0
ICANS	4 (10.3%)	0	0	1 (2.6%)	3 (7.7%)
Fever	29 (74.4%)	5 (12.8%)	17 (43.6%)	7 (17.9%)	0
Exhaustion	12 (30.8%)	9 (23.1%)	3 (7.7%)	0	0
Edema	17 (43.6%)	6 (15.4%)	7 (17.9%)	4 (10.3%)	0
Insomnia	2 (5.1%)	1 (2.6%)	0	1 (2.6%)	0
Dizziness	4 (10.3%)	1 (2.6%)	2 (5.1%)	1 (2.6%)	0
Paresthesia	10 (25.6%)	8 (20.5%)	2 (5.1%)	0	0
Headache	1 (2.6%)	1 (2.6%)	0	0	0
Confusion	2 (5.1%)	0	0	2 (5.1%)	0
Paralysis	2 (5.1%)	0	0	2 (5.1%)	0
Epilepsy	2 (5.1%)	0	0	2 (5.1%)	0
Aphasia	4 (10.3%)	0	0	4 (10.3%)	0
Tremor	1 (2.6%)	0	0	1 (2.6%)	0
Rash	8 (20.5%)	2 (5.1%)	5 (12.8%)	1 (2.6%)	0
Tachycardia	11 (28.2%)	8 (20.5%)	3 (7.7%)	0	0
Atrial Fibrillation	1 (2.6%)	0	0	1 (2.6%)	0
Dyspnea	18 (46.2%)	8 (20.5%)	6 (15.4%)	4 (10.3%)	0
Hypoxia	10 (25.6%)	0	7 (17.9%)	3 (7.7%)	0
Cough	10 (25.6%)	9 (23.1%)	1 (2.6%)	0	0
Expectoration	9 (23.1%)	9 (23.1%)	0	0	0
Hypotension	23 (59.0%)	10 (25.6%)	8 (20.5%)	5 (12.8%)	0
Nausea	16 (41.0%)	14 (35.9%)	2 (5.1%)	0	0
Vomit	8 (20.5%)	7 (17.9%)	1 (2.6%)	0	0
Ventosity	12 (30.8%)	9 (23.1%)	3 (7.7%)	0	0
Diarrhea	18 (46.2%)	12 (30.8%)	3 (7.7%)	3 (7.7%)	0
Abdominal Pain	4 (10.3%)	3 (7.7%)	1 (2.6%)	0	0
Constipation	4 (10.3%)	4 (10.3%)	0	0	0
Granulocytopenia	37 (94.9%)	5 (12.8%)	2 (5.1%)	9 (23.1%)	21 (53.8%)
Lymphocytosis	4 (10.3%)	0	3 (7.7%)	1 (2.6%)	0
Anemia	34 (87.2%)	21 (53.8%)	10 (25.6%)	3 (7.7%)	0
Thrombocytopenia	28 (71.2%)	8 (20.5%)	4 (10.3%)	7 (17.9%)	9 (23.1%)
Hypofibrinogenemia	15 (38.5%)	2 (5.1%)	9 (23.1%)	4 (10.3%)	0
Hypoalbuminemia	11 (28.2%)	6 (15.4%)	5 (12.8%)	0	0
Elevated ALT	8 (20.5%)	6 (15.4%)	1 (2.6%)	1 (2.6%)	0
Elevated AST	8 (20.5%)	7 (17.9%)	1 (2.6%)	0	0

ALT alanine aminotransferasee; AST aspartate aminotransferase; CRS Cytokine Release Syndrome; ICANS Immune Effector Cell-associated Neurotoxicity Syndrome.

29 (74.4%) of 39 patients experienced cytokine release syndrome (CRS); 61.6% were of low grade (grade 1–2). Grade 3 CRS developed in 5 (12.8%) patients. Immune effector cell-associated neurotoxicity syndrome (ICANS) was observed in 4 (10.3%) of 39 patients, which was grade 3 in one patient and grade 4 in three patients (Table 2). The onset and duration of ICANS vary, with duration ranging 4 to 7 days (Supplementary Table S1). In total, 41.0% and 30.8% of the cohort received tocilizumab and glucocorticoids for CRS and ICANS, but no patients needed care in the intensive care unit. In this study, prophylactic anti-infective therapy was not allowed. The patients who suffered ICANS received anti-seizure drugs such as diazepam, propofol, or valproate. Consistent with mild toxicity, serum levels of IL-2, IL-4, IL-6, IL-10, Interferon-gamma (IFN-γ), and Tumor necrosis factor-alpha (TNF-α) were modestly elevated during the first month after CAR-T infusion **(**Supplementary Fig. S4**)**. These data showed that the frequency or severity of AEs of 7 × 19 CAR-T treatment was similar to that of reported anti-CD19 CAR-T cell therapies^1–3,20^, indicating a favorable safety profile.

### Superior efficacy of 7 × 19 CAR-T cells in R/R LBCL

**Fig. 3:**
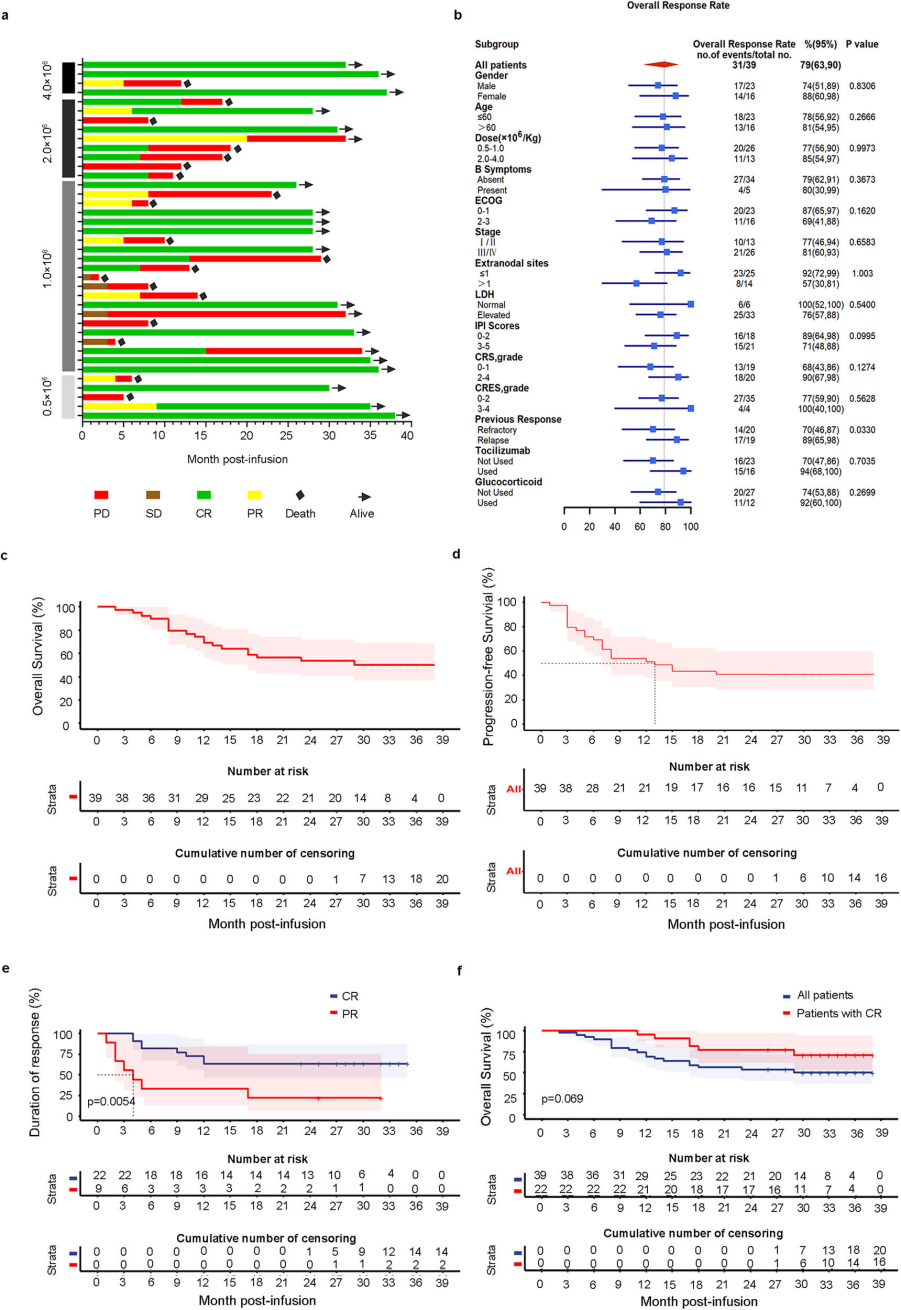
Clinical outcomes and efficacy. **a** Swimmer plot of response duration to 7 × 19 CAR T cell therapy; each bar represents an individual patient. Responses evaluated at month 3 are designated by color (green, CR; yellow, PR; brown, SD; red, PD). Black rhombuses represent death, and bars with black arrows represent patients with an ongoing response. **b** Subgroup analysis of overall response rate evaluated by PET-CT at 3 months after CAR T therapy. **c**, **d** Kaplan-Meier estimates of overall survival (**c**) and progression-free survival (**d**) in patients treated with 7 × 19 CAR T cells. **e** Duration of response in the patients who achieved CR or PR. Significance was calculated using the log-rank test. **f** Overall survival for all enrolled patients and for the patients with CR.

The overall response rate (ORR) was 79.5% (31 of 39), with 56.4% achieving a CR and 23.1% having a PR (Fig. 3a). Interestingly, of the 9 patients with PR at 3 months, 1 patient converted to CR at 6 months and another at 9 months. Thus the best CR rate was 61.5% (95% CI, 44.61 to 76.63). In univariate analysis, patient response rates did not differ across the biological covariates, including subgroups based on dose levels of CAR-T cells. However, it looked like patients with primary refractory diseases had worse responses (Fig. 3b; Supplementary Table S2). As of Nov 30, 2021, 39 patients were followed up for a median of 32 months. The median PFS was 13 months, and median OS was not reached, with estimated rates of 69.2% (95% CI, 56.2% to 85.3%) at 12 months and 53.8% (95% CI, 40.3% to 72.0%) at 24 months (Fig. 3c, d). The median duration of response (DOR) for the patients with CR was not reached, and that of the patients with PR was 4 months (Fig. 3e). Nineteen of thirty-nine patients died from disease progression during the study (Fig. 3a). Among the patients with CR, the estimated 12- and 24-month probabilities of OS were 95.8% (95% CI, 87.1% to 100.0%) and 74.3% (95% CI, 57.8% to 95.6%) respectively (Fig. 3f). Thus, our data indicated that 7 × 19 CAR-T cells produced a potent and durable anti-lymphoma response in patients with R/R LBCL.

### Pharmacodynamics of 7 × 19 CAR-T cells

**Fig. 4:**
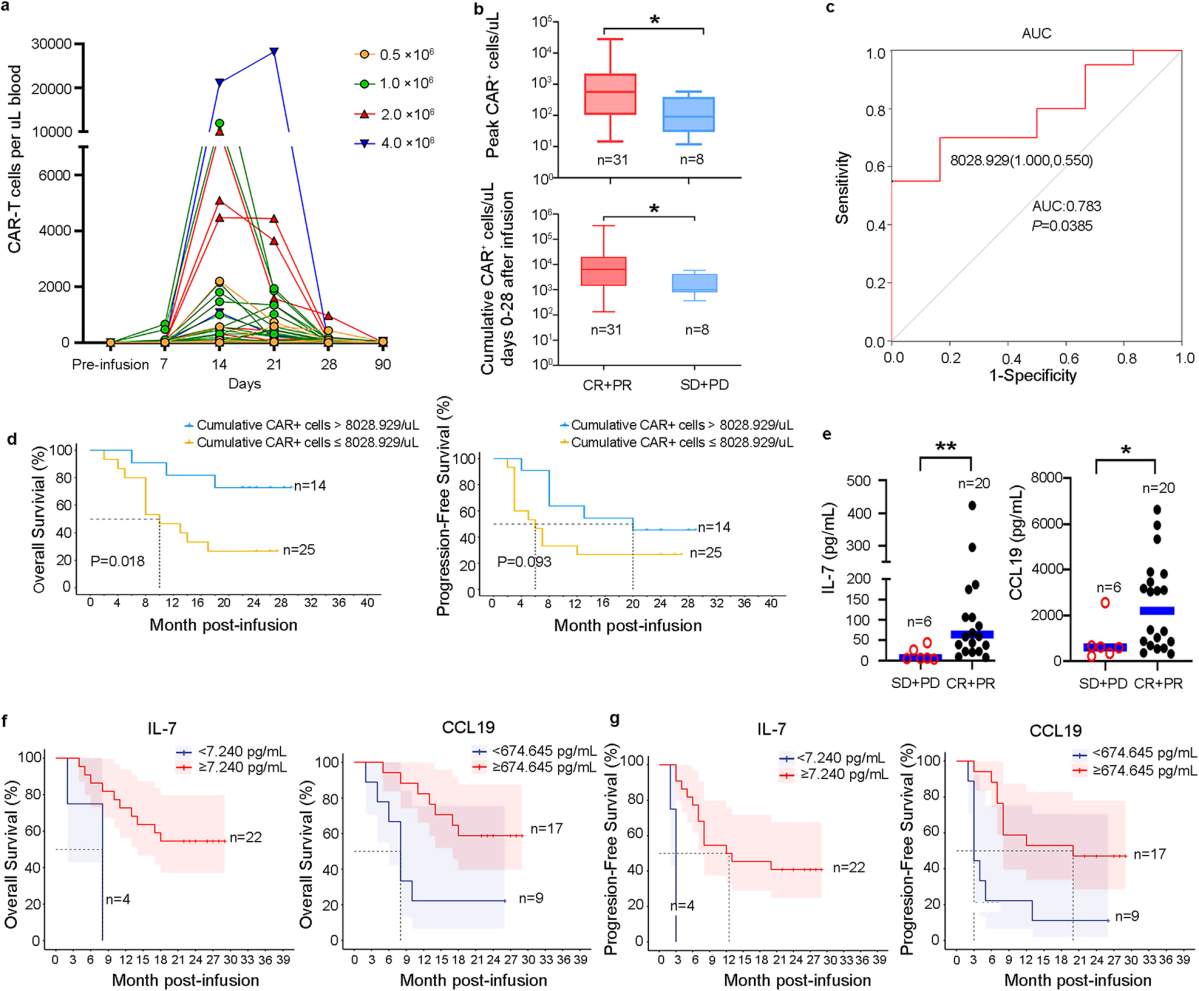
Pharmacodynamics of 7 × 19 CAR T cells and correlation of plasma IL-7 and CCL19 levels with clinical outcomes. **a** CAR T cell kinetics in the peripheral blood of patients treated with different doses of 7 × 19 CAR T cells during the first 90 days after infusion. **b** Bar plots showing the peak number of CAR T cells in the blood of responder (CR + PR) or non-responder (SD + PD) (upper) and the cumulative numbers of CAR T cells in the first 28 days of therapy in the blood of responder and non-responder (lower). **c** Receiver operating characteristic (ROC) curve of cumulative CAR^+^ T cells as predictor for OS and PFS. **d** Kaplan-Meier analysis to estimate OS and PFS according to the cut-off value of cumulative CAR^+^ T cells. **e** Peak values of plasma IL-7 and CCL19 during the first 28 days after CAR T infusion in patients who achieved CR and PR or without response. Kaplan-Meier analysis to estimate peak values of plasma IL-7 and CCL19 during the first 28 days after CAR T treatment as predictors of OS (**f**) and PFS (**g**). The optimal cut-off points for IL-7 and CCL19 were determined by ROC curves and area under the curve. Data are shown as mean ± SD. **a**–**d**, **f**, **g** *n* = 39; **e** *n* = 26. Two-tailed Student’s *t-*test (**b** upper and **e)**, two-tailed Student’s *t-*test with Welch’s correction (**b**, lower), z-test (**c**) and log-rank test (**d**, **f** and **g**). *P < 0.05, **P < 0.01.

We observed substantial CAR-T cell expansion in the blood of patients after CAR-T infusion, which reached a maximal peak concentration in a median time of 14 days (range 8–25) (Fig. 4a). In accordance with an initial expansion of 7 × 19 CAR-T cells, we found concurrent increases in the plasma levels of IL-7 and CCL19 during the first 4 weeks, which correlated with the peak levels of 7 × 19 CAR-T cells, but not with the initial dosages administered (Supplementary Fig. S5). Moreover, the peak concentration and cumulative number of CAR-T cells in the blood of patients during the first 4 weeks were significantly associated with responses (Fig. 4b). However, the cumulative concentrations of CAR-T cells were only associated with better OS, but not with better PFS (Fig. 4c, d), suggesting that other factors may also affect the efficacy of 7 × 19 CAR-T cells. Interestingly, the peak levels of CAR-T in patients were only correlated with grade 2–3 CRS, but not grade ≥ 3 ICANS (Supplementary Fig. S6a).

Given the observed relationship between CAR-T expansion and plasma levels of IL-7 and CCL19, we next asked whether the peak levels of IL-7 and CCL19 are associated with outcomes. Patients who achieved CR or PR showed higher peak levels of IL-7 and CCL19 in the first 4 weeks compared to those without response (Fig. 4e). A receiver operating characteristic curve (ROC) analysis was performed to define the optimal cut-off of IL-7 and CCL19 for response prediction. The optimal cut-off values were 7.24 pg/mL for IL-7 and 674.65 pg/mL for CCL19. Using these cut-off values, we evaluated the relationship between the peak levels of IL-7, CCL19 and OS, PFS, and found that patients with higher peak levels of IL-7 or CCL19 also experienced superior OS and PFS (Fig. 4f, g). These data support the notion that the combination of CAR with IL-7 and CCL19 can enhance anti-tumor potential of CAR-T cells^14–16^, and demonstrate meaningful survival benefit in patients with R/R LBCL. In addition, we detected the expressions of CD127 (IL-7 receptor) and CCR7 (CCL19 receptor) on the surface of 7 × 19 CAR-T cells that were obtained from the peripheral blood of 16 patients after CAR-T treatment, and analyzed the correlation between levels of plasma IL-7 and CCL19 with expressions of CD127 and CCR7, respectively (Supplementary Fig. S6b). The results show that IL-7 and CCL19 receptors were negatively correlated with the concentrations of plasma IL-7 and CCL19, suggesting that 7 × 19 CAR-T cells are susceptible to down-regulation of the cognate cytokine receptor of IL-7 and CCL19.

### Biomarkers are associated with clinical response and toxicity

Serum biomarkers are associated with not only CAR-T therapy efficacy but also CAR-T-associated toxicity^19,22,23^. Thus, we explored the correlation between the levels of cytokines/chemokines and the outcome of 7 × 19 CAR-T treatment. Samples were obtained from 26 patients who signed an informed consent for the use of residual plasma for biomarker research. Results showed that patients with response had higher peak levels of IFN-γ, TNF-α, IL-6, IL-7, IL-13, IL-8, IFN-gamma-inducible protein-10 (IP-10), Macrophage inflammatory protein-1-beta (MIP-1β), Stromal cell-derived factor-1α (SDF-1α), and CCL19 at the first week post-infusion compared to those without response (Supplementary Fig. S7a). However, only higher peak concentrations of IL-7, CCL19, Macrophage inflammatory protein-1-alpha (MIP-1α), MIP-1β, and SDF-1α during the first 4 weeks seemed to correlate with better responses (Supplementary Fig. S7b). Next, ROC analysis was used to define the optimal cut-off values of these cytokines/chemokines that were up-regulated during the first 4 weeks for response prediction (Supplementary Fig. S7c). The results showed that patients who had higher peak levels of IL-7, CCL19, MIP-1α, MIP-1β, and SDF-1α were more likely to achieve better OS and PFS (Supplementary Fig. S7d). We also found that peak levels of 9 cytokines, including IL-6, IL-7, IL-8, Eotaxin, LAG3, B and Tlymphocyte attenuator (BTLA), TNF-α, Monocyte chemoattractant protein (MCP-1), and MIP-1β were significantly elevated in patients with grade 2–3 CRS compared to those with grade 0–1 CRS (Supplementary Fig. S8), suggesting that these biomarkers were correlated with more severe CRS. Because 7 × 19 CAR-T cells possess higher proportions of T_SCM_ and lower proportion of effector T cells (T_EFF_), we analyzed whether these subsets were associated with response rates and found that the patients with response have a higher frequency of naive/T_SCM_ and lower frequency of T_EFF_ in pre-infusion products (Supplementary Fig. S9). These observations are consistent with the fact that T_SCM_ plays a critical role in mediating anti-tumor responses^18,19^.

## Discussion

We report here the safety and efficacy of fourth-generation anti-CD19 CAR-T cells co-expressing IL-7 and CCL19 in patients with R/R LBCL. Our data show no DLT occurring during the DLT observation period. Thus, all patients in both dose-escalation phase and expansion phase (*n* = 39) were assessable for the safety endpoint. In terms of CAR-T specific toxicities, 74.4% of patients experienced CRS, with grade 3 CRS present in 12.8% of patients. None of participants experienced grade 4 CRS. Neurotoxicity developed in 10.3% of patients, which were all grade 3 or grade 4. However, CRS and neurotoxicity completely resolved following supportive care, tocilizumab, or dexamethasone treatment. The frequency of patients with ≥ 3 grade granulocytopenia and thrombocytopenia (79.5 and 41%, respectively) was similar to that in JULIET (81% and 54%) and ZUMA-1 (93% and 58%) study^1,2^. Prolonged or recurrent cytopenias are common following the clinically approved CAR-T therapy for LBCL, which were seen in 15%–37% of patients^24,25^. Meanwhile, the incidence of persistent B-cell aplasia is 25%–38% in patients with B cell lymphoma^25^. With extended follow-up, we also observed that 34.3% (12/35) of patients had neutropenia at 3 months, and B-cell depletion lasted at least 9 months in 18 of the 25 patients. Collectively, the frequency or severity of AEs was comparable to what was observed in the studies of second-generation CAR-T products, indicating a favorable safety profile for the administration of 7 × 19 CAR-T cells.

Pre-clinical study demonstrated potent anti-lymphoma activity of anti-CD19 7 × 19 CAR-T cells, consistent with previously reported anti-CD20 7 × 19 CAR-T^15^. Moreover, our multicenter trial confirmed the superior potency of 7 × 19 CAR-T cells in adult patients with R/R LBCL. At the dosages (0.5–4.0 × 10^6^/kg body weight) used in this study, 31 of 39 treated patients responded to 7 × 19 CAR-T cells (ORR = 79.5%), and 22 patients achieved a CR (56.4%). The median PFS was 13 months, and the median OS was not reached during the follow-up period up to 32 months. A recent study indicated that ECOG PS of 2-–4 and elevated lactate dehydrogenase (LDH) before CAR-T treatment correlated with poor prognosis^26^. Of patients treated with 7 × 19 CAR-T cells in this study, 41% had baseline ECOG PS 2–3, while 84.6% had an elevated LDH. Among the univariate tested, clinical and biologic covariates including ECOG 2–3 and elevated LDH, except for primary refractory diseases, did not associate with treatment response. Taken together, our data demonstrated that 7 × 19 CAR-T cells produced a potent and durable anti-lymphoma response.

The reasons for superior efficacy of 7 × 19 CAR-T cells in B-cell lymphoma are not fully established. IL-7 plays an essential role in expansion and differentiation of naïve T cells into memory stem T cells^11,12,15^, and survival of T cells following repetitive antigen stimulation by upregulating anti-apoptotic molecules such as Bcl-2, Bcl-xl^27^. In vitro, we found that increased proliferation of 7 × 19 CAR-T cells was dependent on secreted IL-7 because the proliferation of CAR-T cells could be reversed by CD127 antibody. In addition, we also observed concurrent increases in the plasma level of IL-7 and CCL19 during the first 4 weeks, which correlated with the peak level of 7 × 19 CAR-T cells. However, the causal relationship between IL-7 and the proliferation of CAR-T cells in vivo is not fully understood. CCL19 can promote the extravasation and directional migration of leukocytes and DCs. Adachi and colleagues demonstrated that anti-CD20 7 × 19 CAR-T cells have increased proliferation, survival, and Tcm/Tscm differentiation upon tumor antigen stimulation in vitro and enhanced T-cell expansion/persistence, tumor-targeting and elimination of pre-established tumor in vivo compared to parental CAR-T cells^15,16^. Interestingly, a clinical trial showed that a higher serum IL-7 peak after CD19 CAR-T treatment was significantly associated with better PFS in the patients who achieved CR^23^. Our data show that patients who achieved CR or PR have higher peak levels of IL-7 and CCL19 during the first month post-infusion compared to those without response, which is associated with superior OS and PFS, thereby highlighting the clinical benefits and therapeutic value of 7 × 19 CAR-T cells.

Higher peak blood CAR-T cell level associated with the efficacy of 7 × 19 CAR-T. This finding was consistent with prior studies of CD19 CAR-T^3,23,28^. Moreover, the patients with ongoing CR had detectable amounts of 7 × 19 CAR-T cells that persisted up to 31 months (Supplementary Table S3). These results, combined with the observation of ongoing CAR-T persistence beyond many years in patients with long-term remissions^29,30^, suggest that increased persistence might contribute to durable remission in lymphoma patients. The serum cytokine/chemokine profile of 7 × 19 CAR-T treatment was similar to that of CD19 CAR-T products. However, we found that patients who had higher peak levels of IL-7, CCL19, MIP-1α, MIP-1β, and SDF-1α during the first 4 weeks seemed to correlate with better responses, and were more likely to achieve better OS and PFS. Considering that MIP-1α, MIP-1β, and SDF-1α are major regulators of leukocyte chemotaxis that evoke an inflammatory response by promoting the activation and transmigration of leukocytes during anti-tumor response^31^, these data indicate that 7 × 19 CAR-T cells are more effective than conventional anti-CD19 CAR-T cells because they elicit not only a specific anti-lymphoma immune response via CAR-mediated tumor antigen recognition but also robust propagation of cytokine/chemokine activation cascades that enhance the persistence/potency of CAR-T and the recruitment/tumor targeting of endogenous immune effector cells such as T cells and DCs, thus facilitating their anti-lymphoma efficacy.

Taken together, CD19-specific 7 × 19 CAR-T cell, an armored CAR-T, demonstrated a favorable toxicity profile and sustained efficacy for relapsed, refractory B-cell lymphoma, supporting 7 × 19 CAR T cells as a promising treatment strategy for B-cell malignancies.

## Materials and methods

### CARs construction

The second generation of the anti-CD19 CAR was designed to contain DNA fragments encoding the following components in-frame from the 5’ to the 3’ end: a CD8α signal peptide sequence and the CD19 binding moiety, i.e., a single chain variable fragment derived from the sequence of FMC63 (GenBank ID: HM852952.1), followed by a CD8α-based hinge, trans-membrane and the 4-1BB and CD3ζ domains. The codon-optimized DNA sequences were assembled into a lentiviral vector backbone (pLenti7.3/V5-DEST Gateway Vector, Thermo Fisher, Massachusetts, USA), of which the CMV promoter was replaced by EF1α promoter. The 7 × 19 CAR was constructed by incorporating another expression cassette containing DNA sequences encoding IL-7 and CCL19 separated by a P2A sequence, under the control of 5 NFAT response elements (REs) and the minimal TATA promoter to facilitate the NFAT-inducible expression of IL-7 and CCL19^17^ in anti-CD19 CAR-T cells.

### Lentivirus production and CAR T cell production for preclinical study

CAR T lentivirus was produced by transient transfection of HEK293T/17 cells (CRL-11268, ATCC, Manassas, USA) with lentiviral plasmid. Briefly, 70% confluent cells were co-transfected via polyethyleneimine (#24765-1, PolyScience) in 150 mm culture dishes with the lentiviral plasmid and the packaging plasmids (Addgene) pMDLg/pRRE (#12251), pRSV-Rev (#12253) and pMD2.G (#12259). The medium was replaced at 24 h and 48 h post-transfection. Viral supernatant was harvested 48 h and 72 h post-transfection and concentrated by ultracentrifugation at 25,000 rpm at 4 °C for 2 h, then stored at –80 °C until use.

Peripheral blood mononuclear cells (PBMCs) were isolated from peripheral blood from healthy donors using Ficoll-Paque density gradient centrifugation and activated with anti-CD3/CD28 Dynabeads (Gibco, Grand Island, NewYork, USA) at 1:1 (bead: cell ratio) in complete AIM-V medium (Gibco) supplemented with 10% fetal bovine serum, 300 IU/mL of recombinant human IL-2 (PeproTech, Cranber, New Jersey, USA), 5 ng/mL IL-7 and IL-15 (Novoprotein, Shanghai, China) for 24 h. After 2 days, the activated T cells were transduced with CAR lentiviral vectors, and then CAR-transduced T cells were proliferated at 0.5 × 10^6^ cells/mL for 10 days.

### Flow cytometry analysis

The expression of anti-CD19 CAR in human T cells was detected by immunofluorescence staining with Alexa Fluor 647-conjugated anti-mouse FMC63 scfv monoclonal antibody (BioSwan Laboratories, Shanghai, China) and flow cytometry analysis. The phenotype of CAR T cells was assessed by immunofluorescence staining and flow cytometry using the following monoclonal antibodies: APC/Cy7-anti-human CD3 (Cat#300426, UCHT1), FITC-anti-human CD4 (Cat#317408, OKT4), PE-anti-human CD8 (Cat#344706, SK1), PE/Cy7-anti-human CD45RA (Cat#304126, HI100) and APC-anti-human CCR7 (Cat#353214, G043H7), Percp/Cy5.5-anti-human CD45RO (Cat#304251, UCHL1), PE/Cy7-anti-human CD197 (CCR7) (Cat#353226, G043H7). PE-anti-human CD95 (Cat#305608, DX2), APC-anti-human CD27 (Cat#302810, O323). The expression of immune checkpoint molecules on CAR T cells was analyzed using PE/Cy7-anti-human PD-1 (Cat#561272, EH12.1) and Brilliant Violet 421 anti-human LAG-3 (Cat#369313, 11C3C65) antibodies. Dendritic cells were analyzed using PE-anti-human CD83 (Cat#305322, HB15e), APC-anti-human HLA-DR (Cat#307610, L243), PE-anti-human CD86 (Cat#305438, IT2.2) and APC-anti-human CD80 (Cat#305220, 2D10) antibodies. Flow cytometric data were acquired with NovoCyte flow cytometer (ACEA Biosciences Inc., San Diego, California, USA), and NovoExpress software was used for flow cytometric data analysis. The fluorophore-antibodies were purchased from BioLegend Inc. (California, USA). The PE/Cy7-anti-human PD-1 antibody was from BD Biosciences.

### Cell lines and in vitro cytotoxicity assay

Raji (human Burkitt’s lymphoma cell, RRID: CVCL_0511), Nalm-6 (human B-cell lymphocyte leukemia cell, CVCL_0092), and K562 (human erythroleukemia cell, CVCL_K562) cell lines were purchased from the American Type Culture Collection (ATCC, Manassas, USA). Jeko-1 (Mantle cell lymphoma cell, CVCL_1865) was purchased from the Cell Bank of the Chinese Academy of Science (Shanghai, China). CD19^+^ K562 cells were established by the transfection of a vector expressing human CD19 antigen. The cells were cultured in RPMI 1640 medium containing 10% FBS and incubated at 37 °C with 5% CO_2_. CD19 CAR and 7 × 19 CAR T cells were stimulated with mitomycin C-treated Raji cells for 7 days and In vitro cytotoxicity assays were carried out using the luciferase-based cytotoxicity assay described previously^32^.

### CAR T-cell proliferation and apoptosis assay

CAR T-cell proliferation was assessed by arboxyfluorescein succinimidyl ester (CFSE) dilution using CellTrace CFSE Cell Proliferation Kit (Invitrogen, California, USA). Briefly, cells (1 × 10^5^) were stained with 1 μM CFSE dye at 37 °C in the dark for 30 min and then co-cultured with mitomycin C-treated Raji cells at the indicated E:T ratio for 24, 48, 72, and 120 h. The dilution of CFSE was evaluated by flow cytometry analysis. The apoptosis of CAR T cells cocultured with mitomycin C-treated Raji cells for 3, 4, and 8 days was assessed by Annexin-V/7-AAD staining (BioGems, Westlake Village, CA) and analyzed by flow cytometry. The plots were gated on CD3^+^ lymphocytes.

### Transwell migration assay

Monocyte-derived DCs were generated by IL-4 and granulocyte-macrophage colony-stimulating factor (GM-CSF, PeproTech) induced differentiation of monocytes isolated from healthy donors. Specifically, PMBCs isolated from blood by Ficoll-Paque density gradient centrifugation were cultured in AIM V medium containing 10% FBS, 100 ng/mL rhGM-CSF, and 20 ng/mL rhIL-4 (PeproTech) for 5 days. 20 ng/mL TNF-α (PeproTech) was added to promote the maturation of DCs for another 24 h culture. Monocyte-derived DCs were harvested on day 7 for phenotypic and migration analyses. The migratory abilities of DCs and T cells in response to the culture medium of CAR T cells were measured using 24-well transwell chambers (Corning) with a polycarbonate filter of 6.5 μm pore-size. Human T cells labeled with CFSE or DCs (5 × 10^6^/mL) were added in the upper chamber, while the supernatant of CAR T cells stimulated with mitomycin C-treated Raji cells for 5 days was added to the lower chamber for the indicated times. The cells migrated from the upper chamber to the lower chamber were determined by flow cytometry analysis. For antibody blocking experiments, DCs or T cells were preincubated for 30 min with anti-CD127 (10 µg/mL, clone A019D5, Biolegend) or anti-CCR7 (5 µg/mL, clone 150503, R&D Systems) antibodies respectively.

### Quantitative PCR (qPCR) analysis

Expression of Bcl-2, Bim and Survivin was quantified with qPCR. Briefly, total RNA was extracted from CAR T cells or control T cells with RNeasy Mini kit (QIAGEN, Redwood City, California, USA). and cDNA was then prepared with PrimeScript RT Regent Kit (TAKARA, Japan). PCR was performed on an Applied Biosystems 7500 real-time PCR system using SYBR Premix Ex Taq (TAKARA, Japan) with following PCR primers: Bcl-2: 5’-ACGACTTCTCCCGCCGCTAC-3’ and 5’-TTGACGCTCTCCACACACAT-3’; Bim: 5’-GTTCTGAGTGTGACCGAGAA-3’ and 5’-CTCCTGTCTTGTGGCTCTGT-3’; survivin: 5’-GGACCACCGCATCTCTACAT-3’ and 5’-GTTCCTCTATGGGGTCGTCA-3’; β-actin: 5′-TTGCCGACAGGATGCAGAA-3’ and 5’-GCCGATCCACACGGAGTACT-3’.

### CD19^+^ human lymphoma xenograft mouse model

All animal studies were performed with the approval of the Institutional Animal Care and Use Committee of Zhejiang University and in compliance with Chinese National Laboratory Animal Guideline for Ethical Review of Animal Welfare. The 6–8-week-old NSG (NOD.Cg-Prkdc^scid^ IL2rg^tm1Wjl^/SzJ) mice (Biocytogen, Beijing, China) were randomized into three groups and injected intravenously with 2 × 10^6^ of luciferase-expressing Nalm-6 cells. The NSG mice were treated by intravenous injection at the E/T ratio of 2.5:1 with anti-CD19 CAR, 7 × 19 CAR T cells or control T cells on day 7 after Nalm6 inoculation respectively. Bioluminescent imaging was performed to assess tumor engraftment using an IVIS® Lumina LT instrument (PerkinElmer, Waltham, Massachusetts, USA).

### Quantification of CAR T cell expansion and trafficking in xenograft mice

To monitor CAR T cells in mice by real-time qPCR. 200 µL peripheral blood was collected by retro-orbital bleeding on day 4, day 12 and day 20. DNA was extracted using QIAamp DNA Blood Mini Kit (QIAGEN) and then amplified with a primer and probe set that is specific for the CD19-CAR. The sequence-specific primers and probes used in animal study were as follows: anti-CD19 CAR forward 5’-TATCGCCACCTATTTCTGCCAG-3’ and reverse 5’-TTTCCTGCAGCTTCACTTCG-3’; probe for anti-CD19 CAR reverse 5’-(FAM)-ACCTTTGGCGGCGGCACCAAGCTGGA-(BHQ1)-3’; β-actin forward 5’-CCACCATGTACCCTGGCATT-3’ and reverse 5’-CGGACTCGTCATACTCCTGC-3’, probe for human β-actin reverse 5’-(HEX)-CCTGGCCTCGCTGTCCACCTTCCA-(BHQ1)-3’. The standard samples were made by a serial 1:2 dilutions of DNA from the infused CAR-T. Based on the percentage of CAR^+^ T cells determined by flow cytometry as mentioned above, the standard curve was obtained from the percentage of CAR^+^ T cells and the cycle threshold value. We then calculated the percentage of CAR^+^ T in the samples from the mice according to the standard curve. All samples were normalized to β-actin. After the CAR^+^ T cells’ ratios were determined, the absolute number of CAR^+^ T cell was calculated by multiplying the percentage of CAR^+^ T by the sum of the total number of infused CAR T cells in the mice.

To monitor CAR T cell trafficking in vivo, anti-CD19 CAR T or 7 × 19 CAR T cells and control human T cells were transfected with a lentiviral vector expressing luciferase-GFP and GFP-luc^+^ cells were purified by FACS sorting to ≥ 98% purity. NSG mice were subcutaneously injected in the left and right flanks with 5 × 10^6^ of Raji cells suspended in 200 µL PBS. When the average tumor volumes reached 100–150 mm^3^, mice were injected intravenously with 1 × 10^7^ of GFP-luc^+^ CD19-CAR and GFP-luc^+^ 7 × 19 CAR T cells. Longitudinal bioluminescent imaging (BLI) was performed to monitor CAR T cell trafficking in vivo.

### Manufacturing and immunophenotyping of CAR T cells for clinical trial

Autologous PBMCs were purified by Ficoll-Paque density-gradient centrifugation from leukapheresis products collected on a COBE Spectra apheresis instrument (Terumo BCT, Lakewood Colorado, USA). Fresh or thawed PBMCs were suspended in AIM V medium (Gibco) with 10% human AB serum (Sigma, St. Louis, MO, USA) and 300 IU/mL IL-2 for incubation in a 37 °C, 5% CO_2_ humidified incubator. After 6 h, adherent cells were removed, and then suspension cells were cultured for 24 h with AIM V medium containing IL-2 (300 IU/mL), 5 ng/mL IL-7 and IL-15 and anti-CD3 and CD28 dynabeads with the ratio of 1: 1. The 7 × 19 CAR lentiviral vector was used to transfect activated T-cells in the presence of 8 μg/mL polybrene at 32 °C with 1200× *g* for 1.5 h, then stopped by resuspending the cells in fresh complete medium supplemented with IL-2, IL-7, and IL-15 for incubation at 37 °C, 5% CO_2_. The expression of CAR on the surface of T cells was assessed on day 5 by flow cytometry analysis and the CAR T cells were harvested on day 11–15. Cell viability was determined by trypan blue exclusion and the frequencies of naïve (CD45RA^+^CCR7^+^), central memory (Tcm: CD45RA^–^CCR7^+^), effector memory (Tem: CD45RA^–^CCR7^–^), and terminally differentiated effector (CD45RA^+^CCR7^–^), CD4^+^ and CD8^+^ T-cell subsets in 7 × 19 CAR T cells were evaluated by immunofluorescence staining with respective fluorochrome-conjugated antibodies and flow cytometric analysis. The median manufacturing time is 13 days (11–15 days). Release criteria for clinical CAR T cell products included the following:

1. Cell viability: ≥ 90%;
2. CD3^+^ cells: ≥ 90%;
3. Endotoxin: ≤ 0.5EU/mL;
4. Mycoplasma: negative;
5. Bacterial culture: negative;
6. Fungal culture: negative;
7. CD3^+^ CAR^+^ T cells: ≥ 10%.

### Study design and oversight of clinical study

This investigator-initiated, open-label, phase 1 and expansion phase study was conducted at multiple centers in China. Eligible patients were aged ≥ 18 years, and had R/R LBCL including histologically confirmed DLBCL, PMBCL, tFL, or MCL; an ECOG PS of 0–3; an absolute neutrophil count of ≥ 1000 per µL; and a platelet count of ≥ 45,000 per µL. A full description of study design and eligibility criteria are provided in the Supplementary protocol. This study was approved by the First Affiliated Hospital, School of Medicine, Zhejiang University Institutional Review Board [2017(3)], and all patients provide the written informed consent in accordance with the Declaration of Helsinki. The study was registered at ClinicalTrials.gov (NCT03258047).

The conditioning regimen consists of intravenous fludarabine (30 mg/m² body-surface area per day) and cyclophosphamide (500 mg/m² body-surface area per day) on days –5, –4, and –3, followed by an infusion of 7 × 19 CAR-T cells on day 0. All patients received lymphodepletion chemotherapy and infusion in the inpatient setting and remained hospitalized until at least 7 days after the CAR-T cell infusion. Any bridging therapy is not allowed. Four escalating-doses that included 0.5 × 10^6^, 1 × 10^6^, 2 × 10^6^, and 4 × 10^6^ CAR^+^ T cells per kg of patient body weight (with an allowance of ± 20%) were tested in the phase 1 study. The dosage used in the expansion phase was determined by the number of CAR-T cells available in the manufactured product (Supplementary Table S4). The maintenance treatment is not allowed for the patients achieved CR and PR after CAR-T infusion unless disease progression is observed during follow-up period. Data are presented until November 30, 2021, and responding patients who were alive entered a long-term follow-up study of up to 5 years (NCT04833504).

### Clinical response assessment

The response was determined by whole-body positron emission tomography/computed tomography (PET/CT) at month 3 according to the Lugano 2014 classification^33^, and then the duration of response was evaluated by ultrasound and CT every 3–6 months until progression.

### Toxicity evaluation

All AEs were monitored from conditioning chemotherapy until one month after CAR-T infusion. Then, only target AEs, which included prolonged or delayed cytopenias, hypogammaglobulinemia, infection, and second malignancies, and serious AEs were recorded until the disease progression. The CRS and ICANS were graded with the American Society of Transplantation and Cellular Therapy Consensus (ASTCT) grading system^34^. Other AEs were evaluated according to the Common Terminology Criteria for AE (CTCAE v. 4.03). CAR-T cells in the blood were monitored by Flow cytometry and PCR analysis enumerating CAR-gene-marked cells. B-cell recovery was also analyzed by Flow cytometry in the patients with ongoing response as described previously^32^.

### Cytokine-release assay

The serum levels of TNF-α, IFN-γ, IL-2, IL-4, IL-6, and IL-10 in patients were assessed using BD™ Cytometric Bead Array Human Th1/Th2/Th17 cytokine kit (BD Biosciences, Franklinlake, New Jersey, USA) by flow cytometric analysis according to the manufacturer’s instructions. The data were analyzed using FCAP Assay V3.0.1 software. The levels of IL-7 and CCL19 in culture supernatants of CAR T cells were assessed using Enzyme-Linked Immunosorbent Assay (ELISA) of IL-7 ELISA kit (ExCell Bio, Suzhou, China) and CCL19 ELISA kit (QuantiCyto, Shenzhen, China).

### Multiplex bead immunoassay

Blood samples from 26 patients were collected before and at several time points during the first 28 days after infusion and plasma samples were collected and stored at –80 °C. The levels of selected cytokine/chemokine/growth factor in the plasma of patients were assessed by multiplex bead immunoassay using 45-ProcartaPlex Human Cytokine/ Chemokine/Growth Factor Panel (Invitrogen). The panel was used to analyze following cytokine and chemokines: MIP-1α, SDF-1α, IL-27, LIF, IL-1, IL-2, IL-4, IL-5, IP-10, IL-6, IL-7, IL-8, IL-10, PIGF-1, Eotaxin, IL-12p70, IL-13, IL-17A, IL-31, IL-1RA, SCF, RANTES, IFN-γ, GM-CSF, TNF-α, HGF, MIP-1β, IFN-α, MCP-1, IL-9, VEGF-D, TNF-β, NGF-β, EGF, BDNF, GRO-α, IL-1α, IL-23, IL-15, IL-18, IL-21, FGF-2, IL-22, PDGF-BB, and VEGF-α, and the 14-ProcartaPlex Human Immuno-Oncology Checkpoint Panel (Invitrogen) was used for immunological factors including TIM-3, CD28, CD137/4-1BB, CD27, CD152/CTLA4, HVEM, IDO, LAG-3, BTLA, GITR, CD80, PD-1, PD-L1, and PD-L2.

### qPCR analysis of CAR T cells expansion and persistence in patients after infusion

To assess CAR T cells in the patient’s peripheral blood, genomic DNA was extracted from PBMCs of patients at multiple time points after CAR T infusion using QIAamp DNA Blood Mini Kit (QIAGEN). The percentage of PBMC CAR^+^ cells was determined by real-time qPCR using anti-CD19 CAR primers and probes as described above. The absolute numbers of CAR^+^ cells in the blood (μL) of patients were calculated by multiplying the percentage of CAR^+^ cells by the sum of the absolute numbers of lymphocytes and monocytes/μL as described previously^32^.

### Peripheral blood B-cell quantification

The B-cell count was determined by immunofluorescence staining and flow cytometric analysis using following antibodies from BioLegend: Pacific Blue-anti-CD45 (Cat#304029), APC/Cy7-anti-human CD3 (Cat#300426), PE-anti-CD19 (Cat#302208), FITC-anti-CD20 (Cat#302304), PE/Cy7-anti-human Ig light chain κ (Cat#316520), and APC-anti-human Ig light chain λ (Cat#316610). Live cells were determined by 7-AAD staining, and normal B-lineage cells were defined as CD45^+^CD3^–^CD19^+^CD20^+^κ^+^λ^+^ lymphocytes. The normal range for blood B cells is 61–321 cells/μL^35^.

### Statistical analysis

Demographic and disease characteristics were analyzed using descriptive statistics and compared between two groups using χ2 test. The probabilities of OS and PFS were estimated using Kaplan-Meier method, and survival curves were compared between groups with a log-rank test. PFS and OS were defined as the time from CAR-T cells infusion to first relapse or death, with censoring at the last follow-up. DOR was defined only for subjects with a response at 3 months and is the time from the first responses to disease progression or death, with censoring at the last follow-up. The ROC curves based on different populations were generated, and the predictive values were then evaluated through examination of the AUC. Specific statistical tests used for in vitro study are described in the figure legends. Analyses were performed using GraphPad Prism version 9 software and R version 4.0.3 software. *P* values < 0.05 were considered significant.

### Supplementary information

Supplementary Information

## Data Availability

Any requests for raw and analyzed data will be reviewed by the Second Affiliated Hospital, School of Medicine, Zhejiang University Institutional Review Board. Patient-related data that are not included in the paper were generated as part of a clinical trial and are subject to patient confidentiality. Any data and materials (e.g., plasma samples or imaging data) that can be shared will need approval from the Second Affiliated Hospital, School of Medicine, Zhejiang University Institutional Review Board, and a Material Transfer Agreement in place.
