# Supplementary material for: Safety and feasibility of anti-CD19 CAR T cells expressing inducible IL-7 and CCL19 in patients with relapsed or refractory large B-cell lymphoma
Source: Cell Discov. 2024 Jan 9;10:5. doi: 10.1038/s41421-023-00625-0 (PMC10774422; doi:10.1038/s41421-023-00625-0)
Supplement: Supplementary file 1 — Supplementary Information [file 41421_2023_625_MOESM1_ESM.pdf]

Supplementary Information

Safety and Feasibility of Anti-CD19 CAR T Cells Expressing Inducible IL-7 and CCL19 in Patients with Relapsed or Refractory Large B-cell Lymphoma

Wen Lei[#], Ai Zhao[#], Hui Liu[#], Chunmei Yang, Cheng Wei, Shanshan Guo, Zhilu Chen, Qunyi Guo, Linjie Li, Mingzhe Zhao, Gongqiang Wu, Guifang Ouyang, Ming Liu, Jinyi Zhang*, Jimin Gao*, Wenbin Qian*

This file includes:

Supplementary Figures S1-19

Figure legends

Supplementary Tables S1, S2, S3, and S4

Supplementary protocol

Supplementary Fig. S1

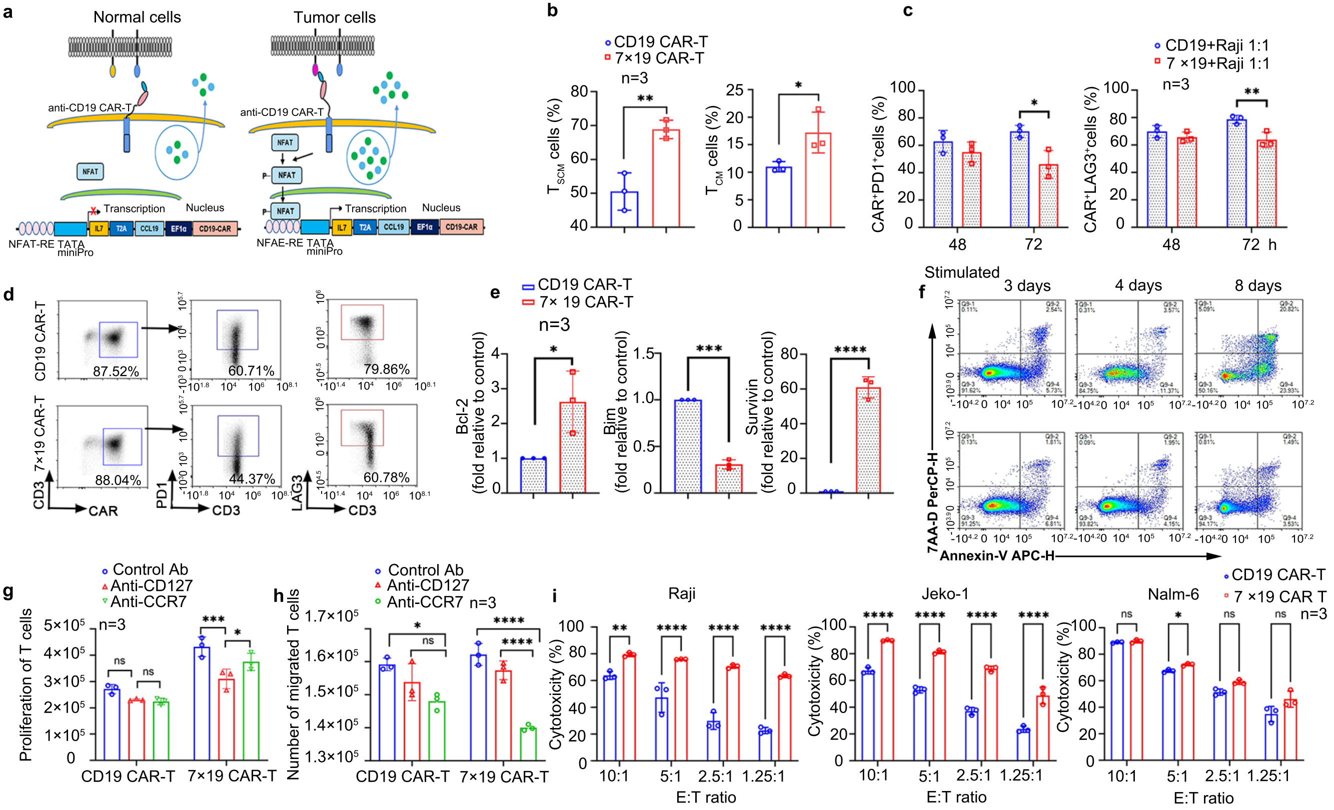

Supplementary Fig. S2

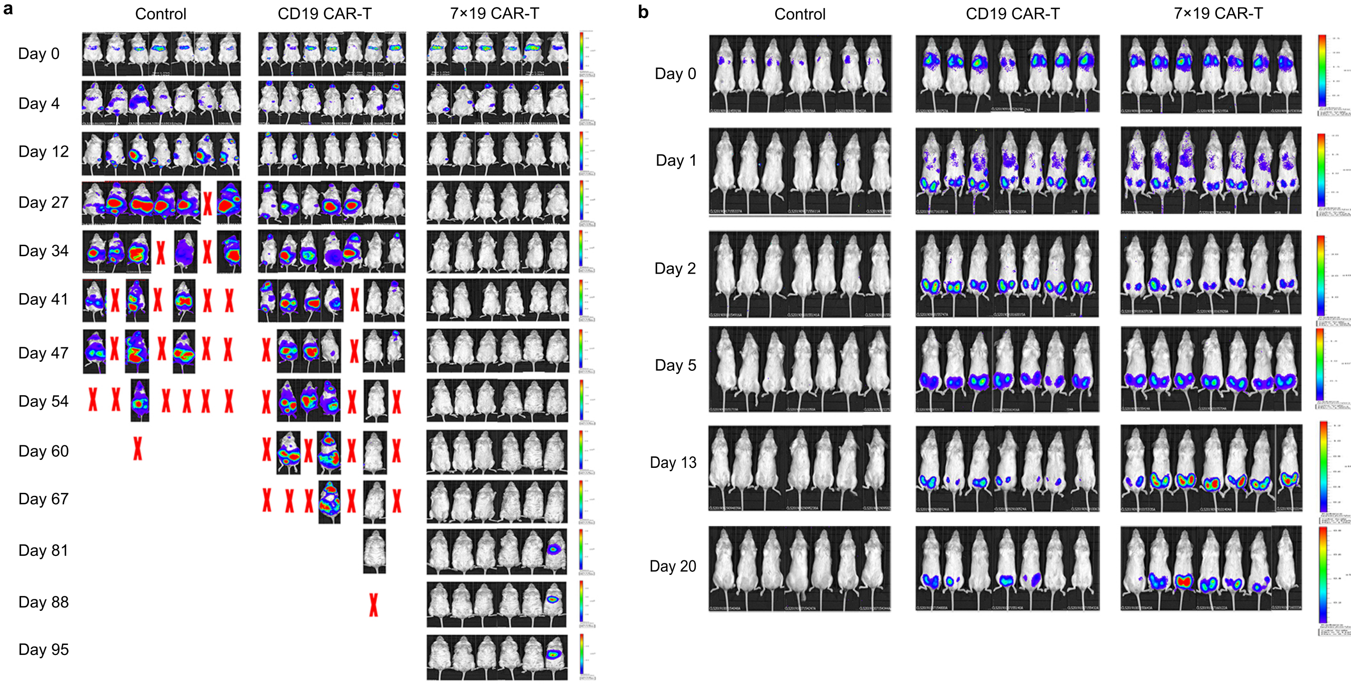

Supplementary Fig. S3

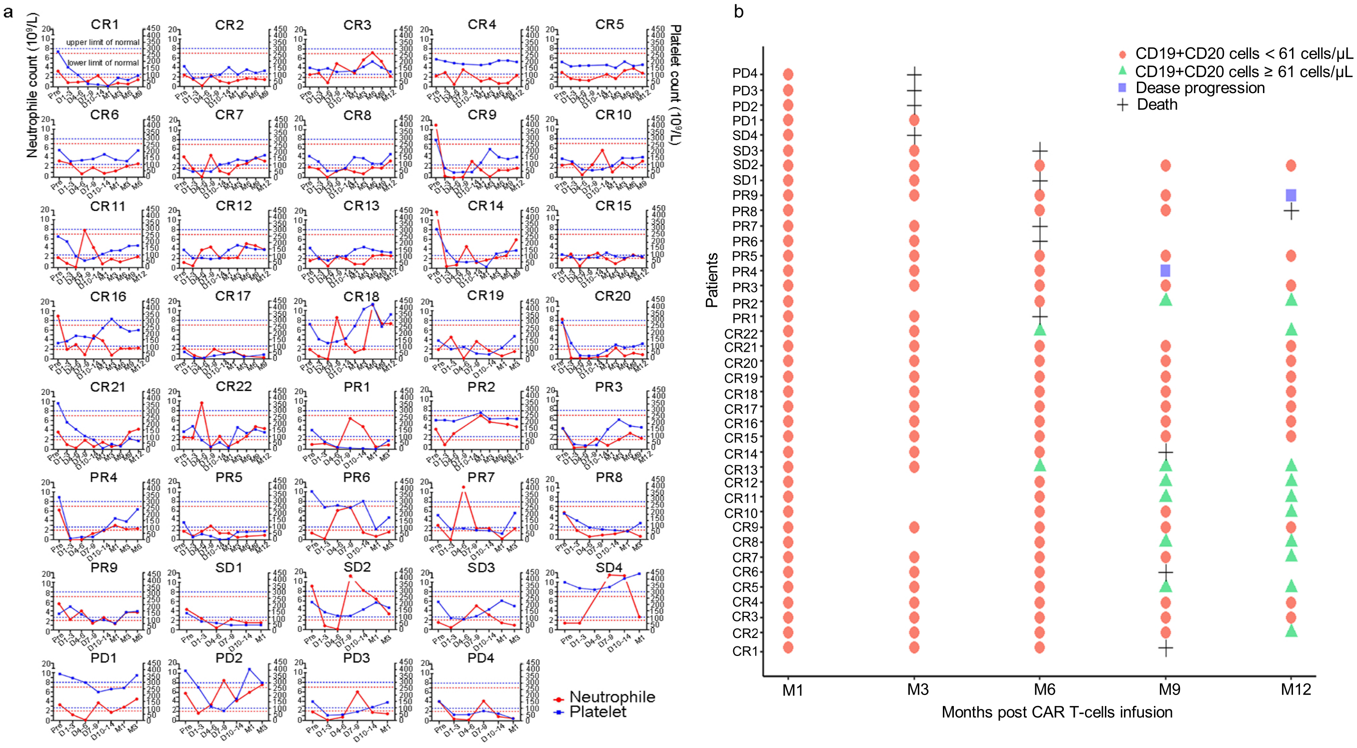

Supplementary Fig. S4

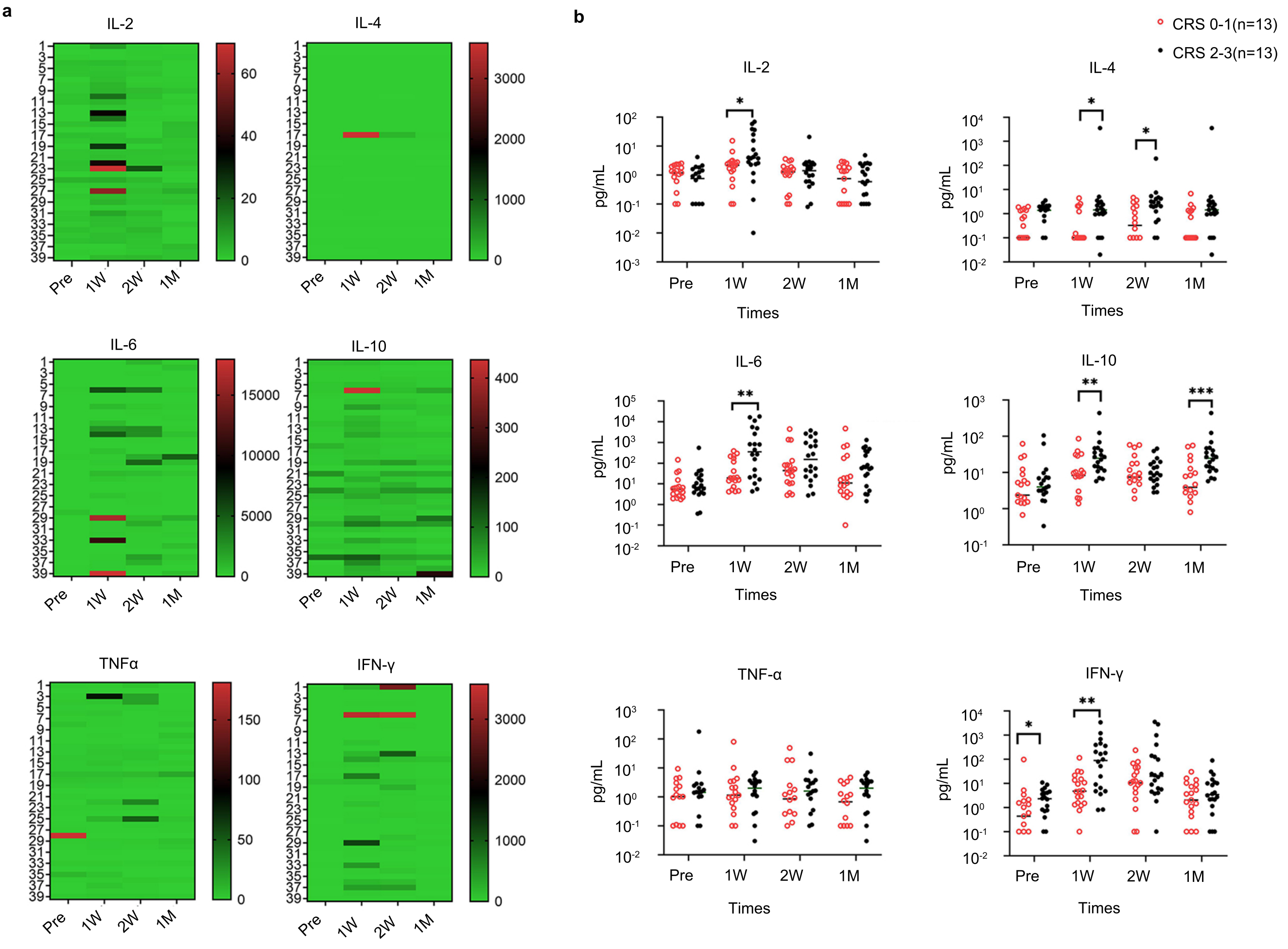

Supplementary Fig. S5

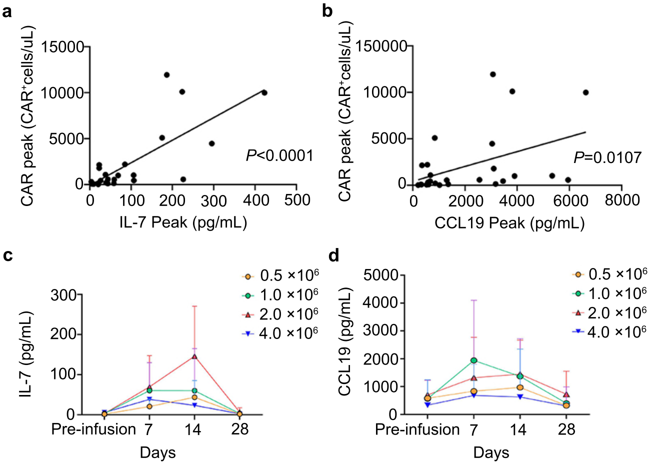

Supplementary Fig. S6

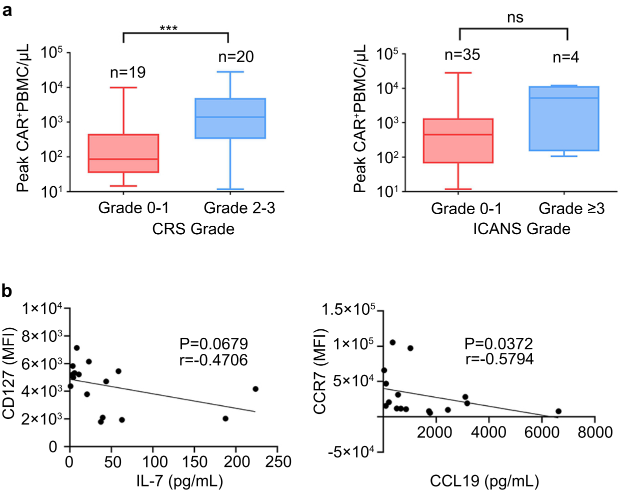

Supplementary Fig. S7

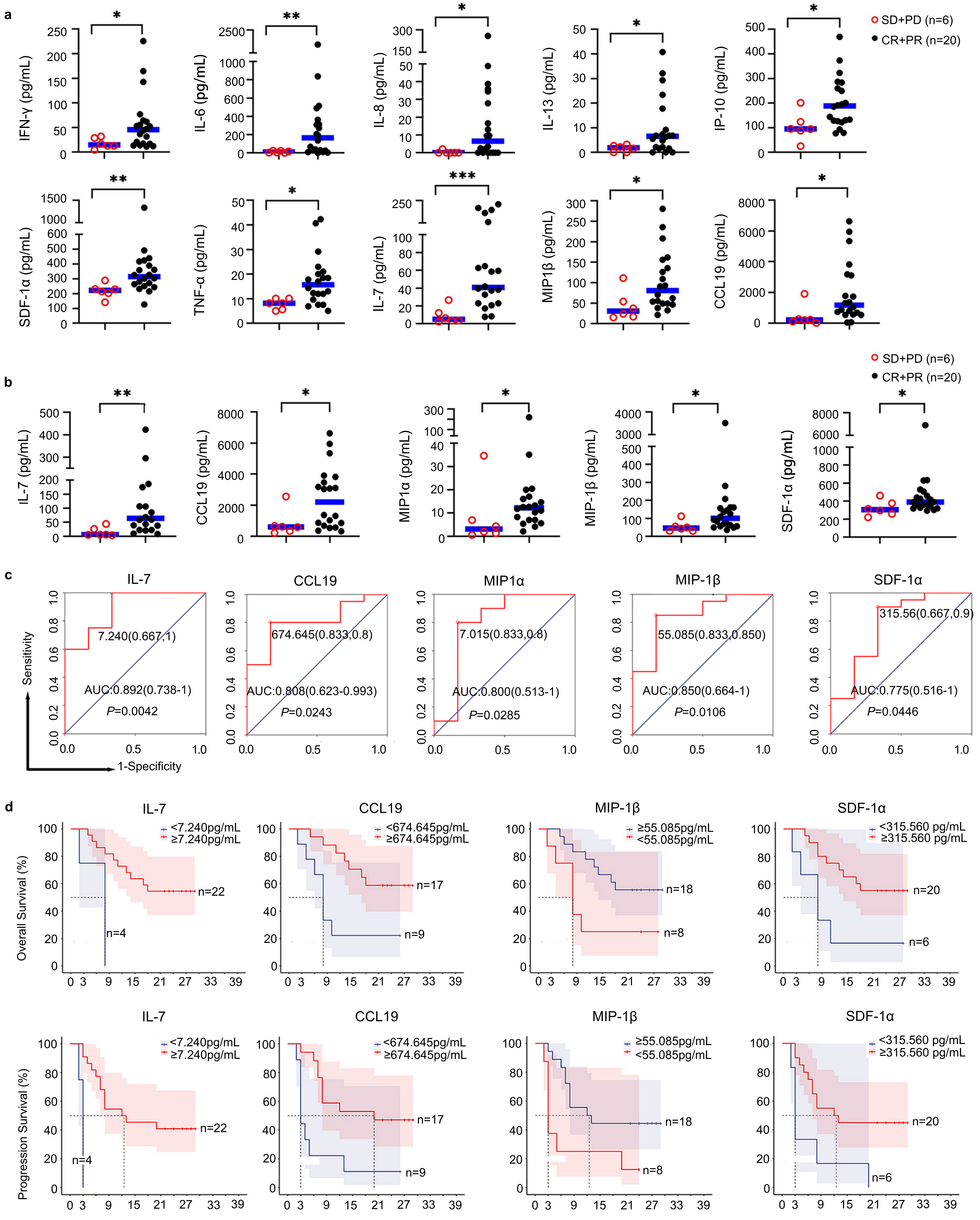

Supplementary Fig. S7

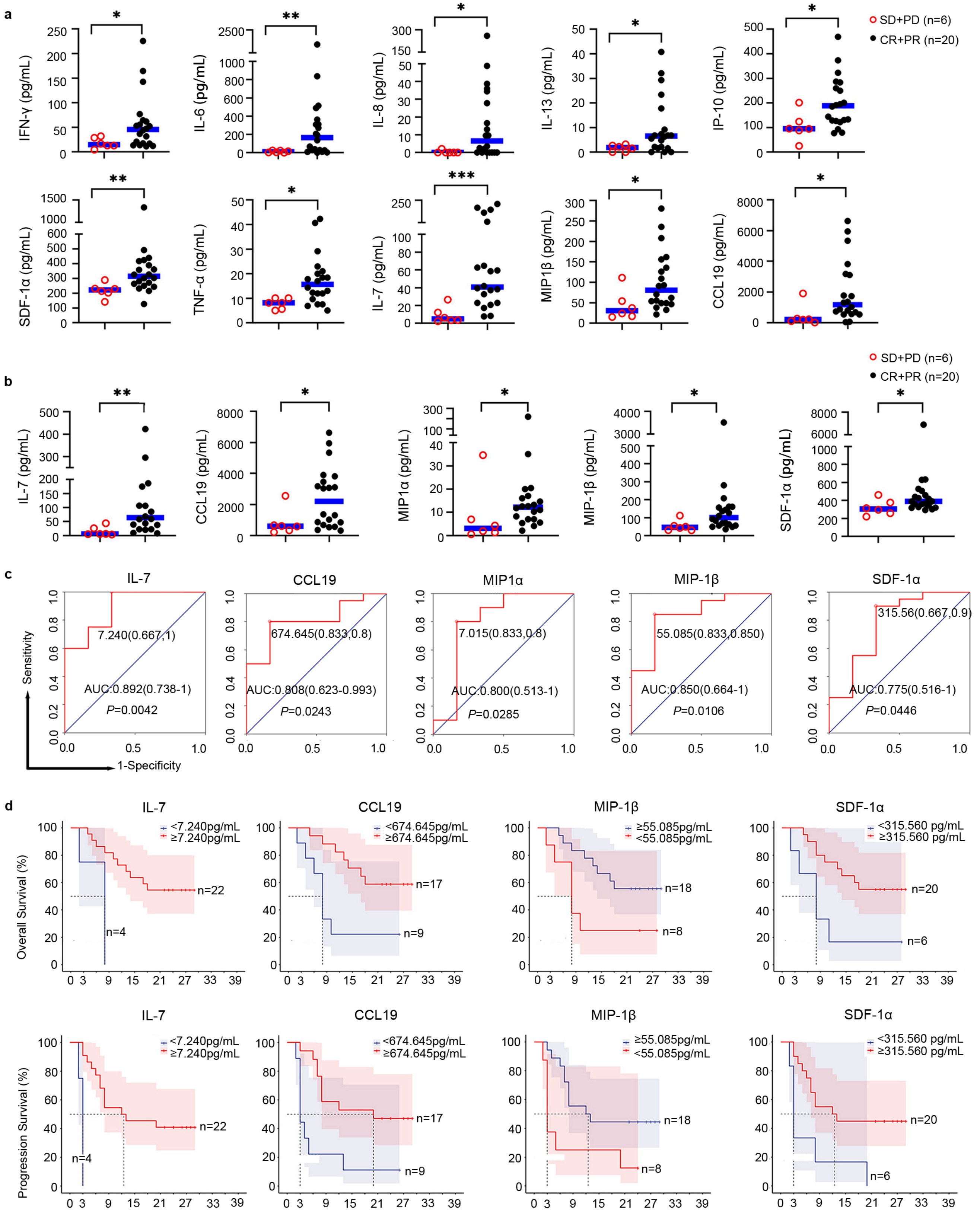

Supplementary Fig. S8

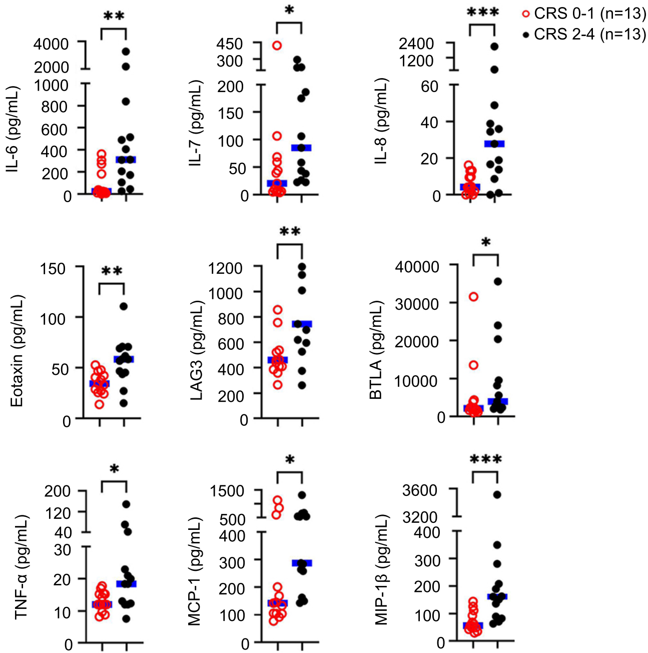

Supplementary Fig. S9

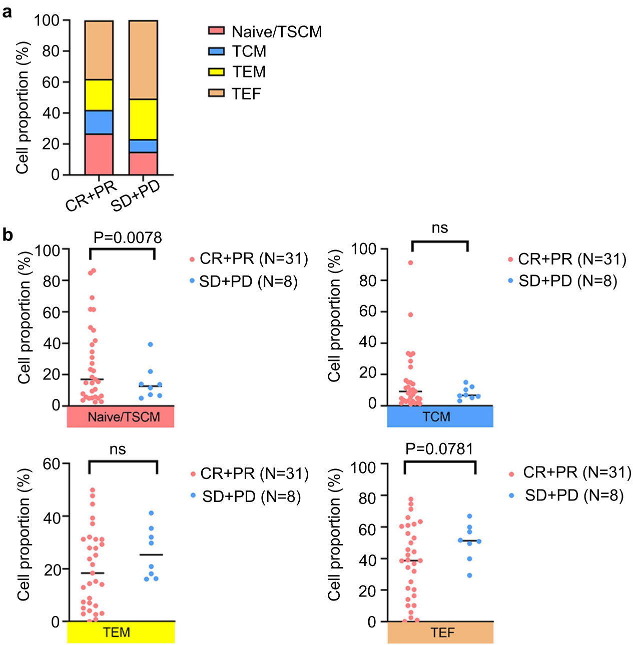

Figure legends

Fig. S1. Functional characterization of 7 × 19 CAR T cells in vitro. a, Schematic diagram showing the CD19-induced NFAT-dependent IL-7 and CCL19 expression in 7 × 19 CAR T cells. **b**, 7 × 19 CAR T cells showed less differentiated phenotypes including Tscm (CD45RA+CD45RO-CCR7+CD95+) and Tcm (CD45RO+CD27+) upon anti-CD3/CD28 antibodies stimulation in vitro. **c, d,** Representative bar graphs (**c**) and FCAS plots (**d**) showing the expression of PD1 and LAG-3 in 7 × 19 CAR T cells co-cultured with Raji cells for the indicated times. The PD-1$^+$ T cells and LAG-3$^+$ T cells were gated in the CD3$^+$CD19$^-$CAR$^+$ T cells. **e**, Expression of Bcl-2, survivin and Bim in 7 × 19 CAR T cells determined by real-time qPCR. **f**, Reduced apoptosis of 7 × 19 CAR T cells stimulated with Raji cell for the indicated times. **g, h,** Anti-CD127 and anti-CCR7 antibody abrogated enhanced proliferation of 7 × 19 CAR T cells (**g**) and 7 × 19 CAR T cell medium-induced T cell migration (**h**). **i**, Enhanced in vitro cytotoxicity of 7 × 19 CAR T cells to CD19$^+$ tumor cells. All data represent three or more independent experiments and are shown as mean ± SD. Two-tailed Student's *t* test (**b** & **e**) and two-way analysis of variance ANOVA (**c** & **g-i**). **P*<0.05, ***P*<0.01, ****P*<0.001, *****P*<0.0001. *ns*: not significant.

Fig. S2. Enhanced anti-tumor activity and expansion of 7 × 19 CAR T cells in NSG xenograft tumor model. a, NSG mouse bearing Nalm6-GFP/Luciferase cells were treated with a single dose (5 × 10^6/mice) of anti-CD19 CAR cells (*n*=7), 7 × 19 CAR cells (*n*=6) or control T cells (*n*=7) on day 7 post-tumor inoculation. Tumor burden was monitored by bioluminescence on IVIS imaging system at the indicated days post CAR T cell infusion. **b,** To monitor CAR T

cells in vivo, anti-CD19, 7 × 19 CAR T cells and control T cells, which expressed luciferase-GFP, were purified by FACS sorting to ≥ 98% purity. NSG mice subcutaneously inoculated with Raji cells (5 × 10^6) in the left and right flanks were injected intravenously with 1 × 10^7 of GFP-luc$^+$ CD19-CAR, 7 × 19 CAR and control T cells (*n*=7), respectively. Longitudinal bioluminescent imaging (BLI) was performed to monitor CAR T cell distribution and expansion in vivo.

Fig. S3. Neutropenia, Thrombocytopenia and B cell aplasia in patients treated with 7 × 19 CAR T cells. a, Peripheral neutrophil and platelet counts before and after infusion of 7 × 19 CAR T cells at the indicated times are shown. Dashed red line represents normal neutrophil counts and dashed black line represents normal platelet count. **b,** B cell aplasia in patients treated with 7 × 19 CAR T cells. B cell numbers in peripheral blood of patients at indicated times post-CAR T cell infusion. Normal range for peripheral blood B cells was 61-321/μL. Complete remission, CR; Partial remission, PR; Stable disease, SD; Progressive disease, PD; Month, M.

Fig. S4. Serum inflammatory cytokines for patients during the first month after CAR T cell infusion. a. Heatmaps show the levels of 6 common inflammatory cytokines in the blood of patients (*n*=39) during the first 30 days after CAR T infusion. **b,** Correlation of the levels of inflammatory cytokines with grade 0-1 and grade 2-3 CRS. Mann-Whitney test was used for *P* values. **P*<0.05, ***P*<0.01.

Fig. S5. Plasma levels of IL-7 and CCL19 are associated with peak level of 7 × 19 CAR T cells, but not with the number of CAR T cells administered. a, b, Correlation of the peak

level of blood CAR^+ cells with the peak levels of IL-7 **(a)** (Spearman correlation r=0.7409; *P*<0.0001) and CCL19 **(b)** (Spearman correlation r=0.492; *P*=0.0107). **c, d,** The plasma levels of IL-7 **(c)** and CCL19 **(d)** in patients received with different dose levels of 7 × 19 CAR T cells during the first 4 weeks. Data are shown as mean ± SD. Data were from 26 enrolled patients.

Fig. S6. The level of 7 × 19 CAR T cells correlated with cytokine release syndrome but not immune effector cell-associated neurotoxicity and the relationship between the levels of cytokines IL-7 and CCL19 and the expression of their receptors. (a) The peak levels of CAR^+ T cells in the blood of patients who had grade 2-3 cytokine release syndrome (CRS) versus those with grade 0-1 CRS **(left)** and patients who had grade ≥ 3 immune effector cell-associated neurotoxicity syndrome (ICANS) versus those with grade 0-1 ICANS **(right)**. Data are shown as mean ± SD (*n*=39). Mann Whitney test. *** *P*<0.001. *ns*: not significant. **(b)** The expressions of CD127 (IL-7 receptor) and CCR7 (CCL19 receptor) on the surface of CAR+ T cells, that were obtained from the peripheral blood of 16 patients after 7 × 19 CAR-T cell infusion, were detected by flow cytometry. The correlations between levels of plasma IL-7 and CCL19 with expressions of CD127 and CCR7, respectively, were analyzed using Spearman correlation.

Fig. S7. Correlation of levels of cytokines/chemokines/growth factors with clinical outcomes. The peak levels of 59 plasma cytokines/chemokines/growth factors in patients were compared between patients who achieved complete remission (CR) or partial remission (PR) and patients who had stable disease (SD) or progressive disease (PD). **a,** Higher levels of IFN-

γ, TNF-α, IL-6, IL-7, IL-13, IL-8, IP-10, MIP-1β, SDF-1α and CCL19 at the first week after CAR T cell infusion were associated with better responses. **b**, Higher levels of IL-7, CCL19, MIP-1α, MIP-1β and SDF-1α during the first 4 weeks after CAR T cell infusion were associated with better responses**.** **c**, Receiver operating characteristic (ROC) curves for the indicated cytokines associated with patient responses. The optimal cytokine concentration cut off values to discriminate subgroups with different progression free and overall survival have been defined by ROC analysis. Area under the ROC curve, AUC. **d**, After optimal cut off values were determined by the ROC curve, survival was estimated by Kaplan-Meier analysis and compared between groups according to the most significantly differentiated cut-off values of IL-7, CCL19, MIP-1β, and SDF-α. Data are shown as mean ± SD (*n*=26). Mann-Whitney test (**a** & **b**), z-test (**c**) and log-rank test (**d**). *P<0.05, **P<0.01, ***P<0.001.

Fig. S8. Correlation of the peak levels of cytokines/chemokines/growth factors in patients treated with 7 × 19 CAR T cells with higher grade cytokine release syndrome. The profiling of 59 cytokines/chemokines in the blood of patients treated with 7 × 19 CAR T cells were measured using multiplexed bead immunoassays. The levels of cytokines/chemokines in patients who developed grade 2-3 CRS were compared with that in patients who had grade 0-1 CRS. Mann-Whitney test (*n*=26). *P<0.05, **P<0.01, ***P<0.001.

Fig. S9. Phenotypic analysis of pre-infusion CAR-T products and the relationship between CAR-T subtypes and the efficacy of 7 × 19 CAR-T cells. The CAR-T cells obtained on two days before infusion were analyzed by flow cytometry to determine the frequencies of

CD45RA+CCR7+ (Naïve/T_{SCM}), CD45RA-CCR7+ (T_{CM}), CD45RA-CCR7+(T_{EFF}) and CD45RA-CCR7-(T_{EM}) subtypes. Target cells were gated in the $CD3^{+}CAR^{+}$ T cells. The difference in frequency of these subtypes between patient with response and patients without response were analyzed using Wilcoxon rank sum tests.

Supplementary Table 1. Characteristics of patients suffered ≥ 3 grade immune effector cell-associated neurotoxicity syndrome

Patient Number	ICANS Grade	Time of onset	Duration (days)	Simultaneous CRS (Grade)	Steroid (doses)	Time of steroid (days)	Tocilizumab	Anti-seizure therapy
1	4	Day 5	6	3	Methylprednisolone, 40 mg q12h × 3 days; Dexamethasone, 10 mg q6h × 4 days	7	Yes	Yes
2	3	Day 3	7	3	Methylprednisolone, 40 mg q12h × 1 day; Dexamethasone, 10 mg q6h × 3 days Dexamethasone, 10 mg q12h × 2 days	6	Yes	Yes
3	4	Day 3	5	3	Dexamethasone, 10 mg q6h × 3 days Dexamethasone, 10 mg q12h × 2 days	5	Yes	Yes
4	4	Day 6	4	2	Methylprednisolone, 40 mg q12h × 2 days; Dexamethasone, 10 mg q6h × 4 days	6	Yes	Yes

Supplementary Table 2. Subgroup analysis of objective response

Patients Characteristics	Numbers	CR/PR at 3 months	PD/SD at 3 months	*P* Value
Patients	39	31	8	
Gender				
Male	23	17	6	0.8306
Female	16	14	2	
Age, y				
≤ 60	23	18	5	0.2666
> 60	16	13	3	
Dose, $\times 10^6$/kg				
0.5-1.0	26	20	6	0.9973
2.0-4.0	13	11	2	
B Symptoms				
Absent	34	27	7	0.3673
Present	5	4	1	
ECOG				
0-1	23	20	3	0.1620
2-3	16	11	5	
Stage				
I / II	13	10	3	0.6583
III/IV	26	21	5	
Extranodal sites, n				
≤ 1	25	23	2	1.003
> 1	14	8	6	
LDH				
Normal	6	6	0	0.54
Elevated	33	25	8	
IPI Score				
0-2	18	16	2	0.0995
3-5	21	15	6	
CRS, grade				
0-1	19	13	6	0.1274
2-4	20	18	2	
ICANS, grade				
0-2	35	27	8	0.5628
3-4	4	4	0	
Previous Response				
Refractory	20	14	6	0.0330
Relapse	19	17	2	
Tocilizumab				

Not used	23	16	7	0.7035
Used	16	15	1	
Glucocorticoid				
Not Used	27	20	7	0.269
Used	12	11	1	

CR, complete remission; PR, partial remission; SD, stable disease; PD, Progressive disease; CRS, cytokine release syndrome; ICANS, immune effector cell-associated neurotoxicity Syndrome; IPI, international prognostic index; LDH, lactate dehydrogenase, ECOG, Eastern Cooperative Oncology Group.CR, Complete remission; PR, Partial remission; SD, Stable disease; PD, Progressive disease.

Supplementary Table 3. Summary of low-level CAR^+ cell persistence and time points

Patient Number	Last time-point after infusion that blood CAR^+ cells were detectable by quantitative PCR^+	Percentages of CAR^+ cell at last time-point	Status
1	CAR^+ cells was still detectable in the 24th month after infusion	0.024%	Ongoing CR
2	CAR^+ cells was still detectable 21 months after infusion	0.004%	Ongoing CR
4	CAR^+ cells was persistently detected 15 months after infusion	0.014%	Ongoing CR
6	CAR^+ cells was still detectable in the 18th month after infusion	0.003%	Ongoing CR
9	CAR^+ cells was still detectable in the 18th month after infusion	0.06%	Ongoing CR
12	CAR^+ cells was persistently detected 31 months after infusion	0.001%	Ongoing CR
18	CAR^+ cells was still detectable 22 months after infusion	0.002%	Ongoing CR
20	CAR^+ cells was still detectable 6 months after infusion	0.028%	Ongoing CR
21	CAR^+ cells was still detectable 26 months after infusion	0.037%	Ongoing CR
25	CAR^+ cells was last detected in the 20th month after infusion	0.003%	Ongoing CR
27	CAR^+ cells was persistently detected 28 months after infusion	0.003%	Ongoing CR
31	CAR^+ cells was still detectable 18 months after infusion	0.024%	Ongoing Response
32	CAR^+ cells was persistently detected 23months after infusion	0.003%	Ongoing CR
34	CAR^+ cells was still detectable 25 months after infusion	30.793%	Ongoing CR
36	CAR^+ cells was last detected in the 27th month after infusion	0.001%	Ongoing CR
38	CAR^+ cells was persistently detected 21 months after infusion	0.143%	Ongoing CR
39	CAR^+ cells was last detected in the 23th month after infusion	0.177%	Ongoing CR

CAR^+ T cell persistence in patients of long-tern remission were monitored by detecting absolute number of CAR^+ peripheral mononuclear cells (PBMCs) in the blood using quantitative RT-PCR. CAR^+ cell levels are presented as a percentage of the total PBMC that contains the CAR gene. CAR gene levels were determined by quantitative PCR.

Supplementary Table 4. Characteristics of enrolled patients and CAR T cell products

Patient	Age /Sex	Diagnosis	Doses (kg)	Culture (day)	Viability (%)	Response	$CD3^+CAR^+$ (%)	$CD3^+$ (%)	$CD3^+CD4^+$ (%)	$CD3^+CD8^+$ (%)	Naïve (%)	Tcm (%)	Tem (%)	TEMRA (%)
1	73/M	MCL	0.35×10^6	15	97.5	CR	22.9	96.6	57.2	23.2	22.47	28.55	27.80	21.18
2	49/F	Non-GCB DLBCL	0.5×10^6	13	98.0	PR	37.6	98.0	73.8	6.1	27.21	1.68	15.22	55.88
3	54/M	Non-GCB DLBCL	0.5×10^6	14	96.0	PD	15.7	93.5	75.1	9.9	22.07	10.30	18.10	49.53
4	60/M	GCB DLBCL	0.5×10^6	15	92.0	CR	25.7	95.4	25.4	64.4	5.87	9.92	31.16	53.05
5	46/F	DLBCL-Unclassified	0.5×10^6	12	96.0	PR	26.7	98.9	63.0	23.23	9.51	32.44	47.74	10.32
6	72/F	Non-GCB DLBCL	1.13×10^6	12	98.0	CR	20.6	98.0	74.9	14.7	69.06	11.47	3.06	16.42
7	67/M	Non-GCB DLBCL	1×10^6	12	96.0	CR	33.1	90.4	41.1	51.3	50.03	3.64	4.07	42.26
8	37/M	Non-GCB DLBCL	1×10^6	12	95.0	SD	37.0	97.2	10.1	82.2	13.83	3.19	16.08	66.90
9	62/F	Non-GCB DLBCL	1×10^6	12	94.0	CR	65.3	98.7	46.2	40.63	48.32	5.56	7.00	39.13
10	69/M	Non-GCB DLBCL	1×10^6	14	92.0	PD	22.3	98.3	10.9	78.8	39.27	15.08	16.25	29.40
11	60/M	Non-GCB DLBCL	1×10^6	13	94.0	SD	28.2	99.1	46.7	36.1	6.70	12.23	41.16	39.91

12	72/M	Non-GCB DLBCL	1×10^6	14	94.0	CR	21.3	95.9	56.9	27.8	34.56	13.92	12.95	38.57
13	55/M	Non-GCB DLBCL	1×10^6	14	97.0	PR	34.6	97.1	15.1	81.6	23.34	11.93	27.93	36.80
14	66/M	GCB DLBCL	1×10^6	14	92.0	SD	30.8	97.9	28.8	58.2	5.07	6.05	32.03	56.85
15	69/F	GCB DLBCL	1×10^6	12	90.0	SD	35.1	94.2	56.2	31.0	11.60	6.97	29.77	51.66
16	50/M	GCB DLBCL	1×10^6	15	91.5	CR	22.6	98.6	53.8	35.2	7.90	16.23	37.13	38.73
17	53/M	MCL	1×10^6	13	92.0	CR	29.1	97.5	56.2	34.8	10.71	58.11	25.19	5.99
18	46/F	Non-GCB DLBCL	1×10^6	14	98.0	CR	38.3	99.0	44.5	33.92	3.72	7.41	44.59	44.27
19	29/M	PMBL	1×10^6	13	98.0	PR	28.6	98.9	58.7	27.16	4.98	2.75	31.22	61.04
20	42/M	Non-GCB DLBCL	1×10^6	13	97.0	CR	16.2	99.5	74.7	14.8	15.53	24.81	39.26	20.41
21	50/F	TFL	1×10^6	14	96.0	CR	61.7	99.3	22.9	65.03	5.17	1.94	18.36	74.52
22	51/M	Non-GCB DLBCL	1×10^6	12	96.0	CR	38.1	99.3	76.8	13.24	5.91	4.45	29.26	60.38
23	55/F	GCB DLBCL	1×10^6	12	98.0	PR	38.1	98.8	90.1	5.97	2.82	3.31	32.09	61.78
24	50/F	GCB DLBCL	1×10^6	12	97.0	PR	32.8	98.0	71.7	14.9	6.44	33.48	49.91	10.17
25	64/F	Non-GCB DLBCL	1×10^6	13	98.0	CR	28.2	99.9	95.0	1.74	5.03	91.31	2.68	0.98
26	57/M	Non-GCB DLBCL	2×10^6	15	93.0	CR	14.1	98.4	13.1	80.3	14.86	4.83	14.37	65.94
27	66/M	GCB DLBCL	1.43×10^6	14	95.0	CR	11.0	95.0	13.0	69.0	14.72	1.68	5.97	77.64
28	37/M	GCB DLBCL	1.8×10^6	11	91.4	PD	12.0	98.5	29.9	65.0	14.05	5.24	20.85	59.86

29	62/F	Non-GCB DLBCL	$2×10^6$	12	97.0	CR	32.4	98.0	43.4	40.1	61.63	1.09	2.89	34.39
30	68/M	Non-GCB DLBCL	$2×10^6$	12	93.0	CR	52.4	98.7	62.4	23.7	39.12	14.85	14.07	31.96
31	65/M	MCL	$2×10^6$	13	96.0	PR	38.1	99.5	34.8	48.0	30.94	33.34	21.61	14.11
32	44/M	Non-GCB DLBCL	$2×10^6$	14	98.0	CR	23.0	90.2	5.5	88.0	84.68	15.12	0.01	0.19
33	46/M	HGBL-DHL	$2×10^6$	13	91.0	PD	31.5	94.9	4.4	79.2	7.32	6.45	35.37	50.86
34	69/F	Non-GCB DLBCL	$2×10^6$	13	93.0	PR	37.14	99.36	73.95	18.06	17.06	9.24	23.74	49.96
35	64/M	Non-GCB DLBCL	$2×10^6$	15	94.0	CR	39.9	98.7	47.7	26.7	2.51	2.86	31.23	63.40
36	52/F	GCB DLBCL	$4×10^6$	15	95.0	CR	15.7	93.0	29.6	59.7	86.33	10.35	0.75	2.58
37	65/M	Non-GCB DLBCL	$4×10^6$	12	96.5	PR	48.1	98.0	47.5	44.5	18.48	1.40	8.81	71.31
38	48/F	t-FL	$4.3×10^6$	15	96.0	CR	12.6	92.0	54.4	28.7	61.48	8.17	5.09	25.25
39	50/F	Non-GCB DLBCL	$4×10^6$	13	95.0	CR	41.8	99.5	73.7	10.6	41.71	5.03	7.37	45.62

GCB,Germinal center B cell; DLBCL, Diffuse large B-cell lymphoma; HGBL-DHL, High grade B-Cell lymphoma; MCL, Mantle cell lymphoma; t-FL:Transformed foliicular lymphoma; PMBL, Primary mediastinal large B-cell lymphoma; CR, Complete remission; PR, Partial remission; SD, Stable disease; P, Progressive disease; Tcm, Central memory T cells; Tem: Effectively memory T cells; TEMRA, Terminally differentiated effector memory T cells.

Supplementary protocol

This appendix has been provided by the authors to give readers additional information about our study.

Supplement to: Safety and Feasibility of Anti-CD19 CAR T Cells Expressing Inducible IL-7 and CCL19 in Patients with Relapsed or Refractory Large B-cell Lymphoma.

Wen L, et al.

Contents

Methods ... **3**

1 Full listing of study sites ... 3

2 Protocol ... 4

2.1 Introduction ... 4

2.2 Study Design ... 4

2.3 Study Objectives ... 4

2.4 Study Endpoint ... 5

2.5 Study Diagram ... 6

2.6 Study Procedure ... 10

2.7 Study Withdrawal ... 20

2.8 Suspension Criteria ... 20

2.9 Study Population ... 21

2.10 Number of Subjects ... 21

2.11 Study Eligibility ... 23

2.12 Exploratory ... 26

2.13 Covariates ... 26

2.14 Clinical efficacy evaluation ... 27

2.15 Adverse Events ... 28

2.16 Laboratory Test ... 43

2.17 Statistical Procedures ... 45

Reference ... 46

Methods

1 Full listing of study sites

Centers	Lead Investigator	No.Patients
Department of Hematology, The First Affiliated Hospital of Zhejiang University, Hangzhou, Zhejiang, China	Wenbin Qian	24
Department of Hematology, Tongde Hospital of Zhejiang Province, Hangzhou, Zhejiang, China.	Zhilu Chen	4
Department of Hematology, Taizhou Hospital of Zhejiang Province, Wenzhou Medical University, Linhai, Zhejiang, China	Qunyi Guo	2
Department of Hematology, Lishui Municipal Central Hospital, Lishui, Zhejiang, China	Linjie Li	2
Department of Hematology, Jinhua Municipal Central Hospital, Jinhua, Zhejiang, China	Mingzhe Zhao	2
Department of Hematology, Dongyang People's Hospital, Wenzhou Medical University, Dongyang, Zhejiang, China	Gongqiang Wu	2
Ningbo Clinical Research Center for Hematological Tumor Diseases, Department of hematology, the First Affiliated Hospital of Ningbo University, Ningbo, Zhejiang, China	Guifang Ouyang	2
Department of Hematology, First Affiliated Hospital of Zhejiang Chinese Medical University, Hangzhou, China	Junfa Chen	1

2 Protocol

2.1 Introduction

This is an investigator-initiated, open-label, phase 1 and expansion phase study evaluating the safety and efficacy of CD19-specific 7 × 19 CAR-T cells in subjects with refractory/relapsed (R/R) aggressive B-cell lymphoma. The clinical trial will be separated into two parts designated as phase 1 and expansion phase.

2.2 Study Design

The study consisted of seven steps: (1) screening, (2) leukapharesis, (3) manufacturing of CAR-T cells, (4) conditioning chemotherapy treatment, (5) investigatory CAR-T product treatment, (6) post treatment assessment, and (7) long-term follow-up.

2.3 Study Objectives

There are two parts to this study.

The primary objective of the dose escalation phase of the study (Part A) is to determine the maximally tolerated dose (MTD) of CD19-specitific 7 × 19 CAR-T cells in subjects with R/R large B-cell lymphoma (LBCL).

The primary objective of the expansion part of the study (Part B) is to validate the safety.

The secondary objective of this study is to evaluate efficacy of CD19-specitific 7 × 19 CAR-T in subjects with R/R LBCL.

The exploratory objectives of this study include:

- Determine the overall survival (OS) and progression-free survival (PFS) of subjects treated with 7 × 19 CAR-T cells
- Evaluate the expansion and persistence of 7 × 19 CAR-T cells in the blood
- Detect plasma levels of cytokine/chemokine in the blood of subjects after treatment of 7 × 19 CAR-T cells
- Evaluate the levels of normal B-cells in blood

2.4 Study Endpoint

The primary endpoints of the study are incidence and severity of adverse events (AEs) related to the CAR-T cell treatment, including dose-limiting toxicities (DLTs).

The secondary endpoints of this study are, but not limited to treatment response that include complete response (CR) and partial response (PR), which will be determined by whole-body PET-CT at month 3 according to the Lugano 2014 classification[1] ;

The exploratory endpoints of the study include:

- OS
- PFS
- Quantification of level of CAR^+ T cell in blood over time
- Quantification of level of normal B-cells over time.
- Assessment of cytokine/chemokine levels in blood of subjects treated with 7 × 19 CAR-T cells as it correlates to clinical response and CAR-T related toxicity.

2.5 Study Diagram

The study is a multicenter, open-label, signal-arm clinical trial to evaluate the safety and efficacy of 7 × 19 CAR T cells in adult patients with R/R LBCL. A schematic of the study design depicts 2 parts: Part A (Dose escalation phase) and Part B (Expansion phase).

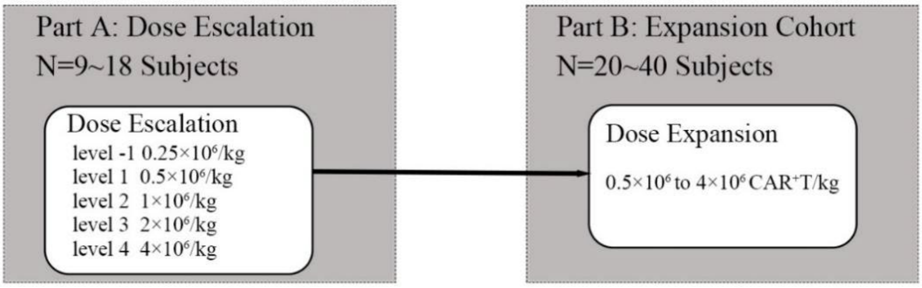

Figure 1 Schematic of Study Design

Part A: Dose Escalation, Overview

Up to approximately 12-18 adults with $CD19^+$ R/R LBCL will be enrolled using a 3 + 3 dose escalation approach:

(1) 3 patients treated per dose level;

(2) If no DLT, dose is escalated for the next cohort of 3 patients;

(3) If 2 DLT in dose level 1, a new dose level -1 will be added in the trail; If ≥2 DLTs in the dose level -1, this clinical trail will be stopped;

(4) If 1 DLT, 3 additional patients are treated at this level with dose escalation only if no additional DLTs; If ≥2 DLTs, prior dose level is defined as MTD;

(5) MTD decided when 6 patients are treated at a dose level with <2 DLTs;

(6) Dose escalation will not proceed until the appropriate number of subjects at that dose level have met the requirement for MTD determination, which includes a minimum of 28 days of

follow-up post 7 × 19 CAR T infusion for DLT determination. A recommended dose may be determined without determining an MTD. The schematic of 3 + 3 dose escalation program as follows:

Figure 2 The schematic of 3+3 dose escalation program

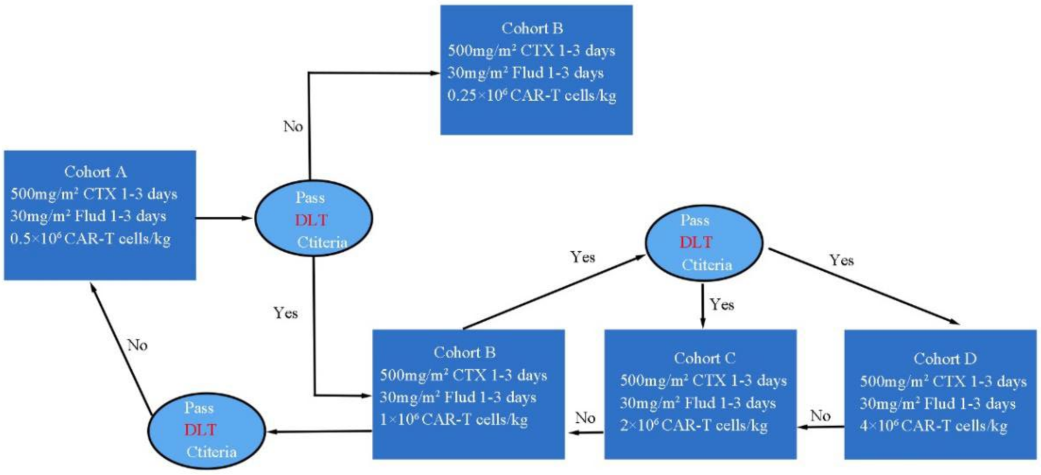

Table 2-1. Dose Escalation Levels

Dose Level	CAR⁺ T cells/kg (± 20%)[a]
-1	$0.25×10^6$
1 (Starting Dose)	$0.5×10^6$
2	$1×10^6$
3	$2×10^6$
4	$4×10^6$

[a] 7 × 19 CAR-T dose is expressed as the number of anti-CD19 CAR^+ T cells per kilogram.

➢ **Maximally Tolerated Dose Definition**

The MTD is the highest dose that causes DLTs in <2 of 6 subjects. For a dose level to be declared the MTD, at least 5 evaluable subjects must be enrolled with no DLTs reported, or 6 evaluable subjects if 1 subject experience a DLT.

A MTD may not be determined in this study. A decision to move to expansion cohorts using the recommended dose of expansion phase may be made in the absence of a MTD provided the dose is at or below the maximum dose studied in Part A of the study.

- **Dose-Limiting Toxicity Definition**

DLT is defined as any 7 × 19 CAR T-related Grade 3 to 5 toxicity occurring within the first 28 days following infusion of the CAR-T cell product, with the following exception:

- Grade 3 Cytokine Release Syndrome (CRS) that responds to appropriate medical intervention within 3 days. (recovers to ≤ Grade 2);
- Aphasia/dysphasia or confusion/cognitive disturbance which resolves to grade 1 or less within 2 weeks and to baseline within 4 weeks;
- Grade 3 to 4 Tumor Lysis Syndrome (TLS) lasting < 7 days;
- Hematologic toxicities;
    - Grade 3 neutropenia of any duration or Grade 4 neutropenia lasting < 14 days;
    - Grade 3 anemia of any duration or Grade 4 anemia lasting < 14 days;
    - Grade 3 thrombocytopenia of any duration or Grade 4 thrombocytopenia lasting < 21 days;
    - All cytopenias except neutropenia, anemia, and thrombocytopenia as described above;
- Non-hematologic toxicities:

- Fever of any grade, including febrile neutropenia;
- Grade 3 diarrhea lasting < 72 h;
- Grade 3 nausea and/or vomiting lasting < 72 h;
- Grade 3 fatigue lasting < 7 days;
- Grade 3 to 4 transaminase, bilirubin, creatinine kinase, blood urea nitrogen (BUN), or creatinine elevation lasting < 7 days;
- Asymptomatic lipase elevation in the absence of any clinical signs or symptoms of pancreatitis;
- Any non-hematologic Grade 3 clinical laboratory AE that is asymptomatic and rapidly reversible (returns to baseline or to ≤ Grade 2 within 7 days).

The National Cancer Institute Common Terminology Criteria for Adverse Events (NCI-CTCAE) Version 4.0 will be used to grade toxicities during the trial unless specified above.

Part B: Expansion Phase

The planned dose used in expansion phase is based on the review of the preliminary safety and efficacy data generated in Part A of the study. In the latest protocol version, the dose of CAR-T cells used in the expansion phase was determined by the number of CAR T cells available in the manufactured product, which include four dose level from 0.5×10^6 to 4×10^6 CAR^+ T cells per kg.

2.6 Study Procedure

Every subject will undergo the following procedure: Screening period; Leukapharesis and CAR-T cells production; Conditioning chemotherapy treatment; Investigational 7 × 19 CAR-T product treatment; Post treatment assessment; Long term follow-up.

- **Screening Period**

During the screening period, the researchers will collect the patient's medical history to evaluate the subject's eligibility and inform the treatment process and the potential risk. Informed consent should be signed by the subject before enrollment. Blood samples were collected and tested for evaluation of HIV infection and the number of $CD3^+$ T cells in blood.

- **Leukapharesis and CAR-T cells production**

Peripheral blood mononuclear cells (PBMCs) were harvested by leukapheresis and transported to the CAR-T cells production laboratory (Academic GMP Laboratory of Zhejiang University). During 12-14 days, T cells undergo the followed periods, including the activation, transduction, proliferation, and quality controls before infusion; the details are as follows:

Autologous PBMCs were collected on a COBE Spectra apheresis instrument (Terumo BCT, Lakewood Colorado, USA). Fresh or thawed PBMCs were suspended in AIM V medium (Gibco, Grand Island, NewYork, USA) with 10% human AB serum (Sigma, St. Louis, MO, USA) and 300 international units (IU)/mL interleukin-2 (IL-2) for incubation in a 37 °C, 5% CO2 humidified incubator. After 6 h, adherent cells were removed, and then suspension cells were cultured for 24 h with AIM V medium containing IL-2 (300 IU/mL), 5 ng/mL IL-7 and IL-15 (Prime Gene, Shanghai, China), as well as anti-CD3 and CD28 dynabeads (Gibco) with the ratio of 1: 1 for activating T cells. Next, 7 × 19 CAR vector were used to infect the activated T-cells with 8 μg/mL polybrene to increase viral transduction. The transduction process was performed under the condition of 1200g, 32 °C for 1.5 h and then stopped by discarding the excess uninfected virus and resuspending the cells in fresh complete medium supplemented with IL-2, IL-7, and IL-15. The expression of CAR on the surface of recombinant CAR-T cells was detected by flow cytometry on day 5. The CAR-T cell products were harvested on day 13-15. Release tests of CAR-T cell products manufactured under GMP condition include cell viability, endotoxin, potency, stability, and microbiological testing.

<u>Release criteria for CAR T cell products include the following:</u>

(1) Cell viability: ≥ 90%;

(2) CD3+ cells: ≥ 90%;

(3) Endotoxin: ≤ 0.5 EU/mL;

(4) Mycoplasma: negative;

(5) Bacterial culture: negative;

(6) Fungal culture: negative;

(7) CD3+ CAR+ T cells: ≥ 10%.

- **Conditioning Chemotherapy Treatment**

The 7 × 19 CAR-T cells should be administrated after a conditional chemotherapy regimen consisting of fludarabine 30 mg/m^2/day and cyclophosphamide 500 mg/m^2/day, consistently for 3 days.

Table 2-2. Lymphodepletion Treatment Plan

**Drug**	**Dose**	**Day**
Cyclophosphamide	500 mg/m^2/day IV infusion over 30 min	-5,-4, and -3
Fludarabine	30 mg/m^2/day IV infusion over 30 min administered immediately after Cyclophosphamide (Fludarabine dose should be reduced based on renal function) *	-5,-4, and -3

*Adult subjects with moderate impairment of renal function (creatinine clearance 30 to 70 mL/min/1.73 m^2) should have a 20% dose reduction of Fludarabine Phosphate Injection, USP. http://dailymed.nlm.nih.gov/dailymed/drugInfo.cfm?setid=cf5255cc-91fd-4132-973b 764dba142eae.

- **CAR-T cells infusion**

The 7 × 19 CAR-T cells were infused two days after the end of chemotherapy (day 0). CAR-T cells will be administered as a single intravenously administration. The entire return process should be within 30 minutes. Vitals signs are to be monitored prior to CAR-T infusion, upon completion of the infusion, and every hour for the next 4 h. Infusion reaction, including

anaphylaxis, will be managed according to the medical judgment of the physician overseeing the infusion.

- **Post-Treatment Assessment**

The CAR-T treatment period begins the day of the first CAR-T cells infusion up through and including 30 days after infusion. During this period, researches need to monitor the temperature, if the temperature ≥38 °C, the physicians should evaluate for symptoms of CRS and the infectious. Blood cultures, urine culture, chest X-ray, and cytokines are required to be tested for evaluating the AEs.

- **Follow-Up**

Subjects were assessed according to the plan. We will evaluate the therapeutic effect every four weeks and every three months after 3 months. After a close follow-up of 3 months, the subjects will undergo a semi-annual medical history assessment, physical examination, and blood examination until disease progression. After this assessment, participants will enter a three-year annual follow-up to assess long-term health problems such as recurrence of malignant tumors.

Figure 3 Summary of Treatments to be Performed or Administered

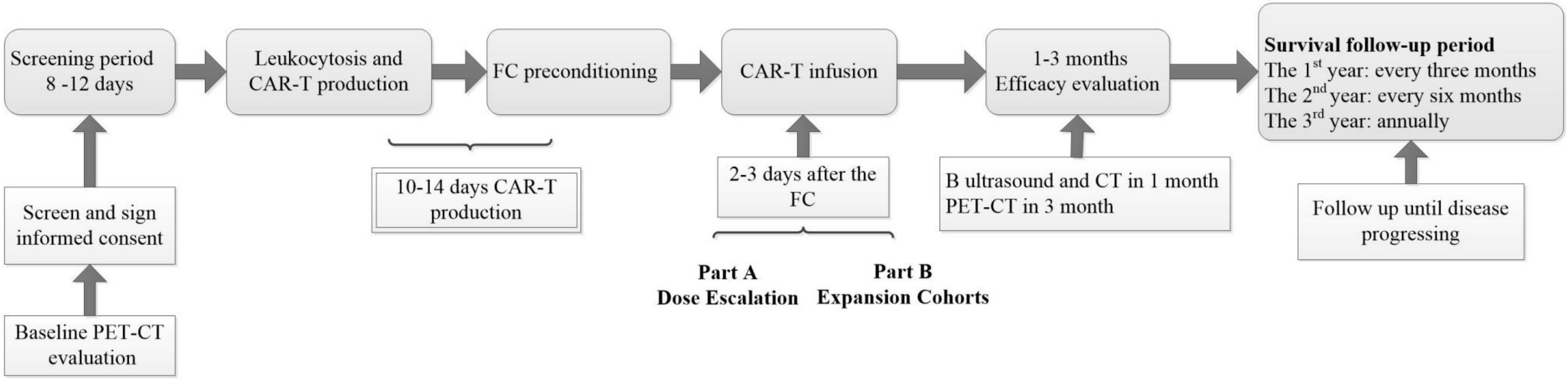

Table 2-3. Schedule of Subjects Screening Period, 7 × 19 CAR-T Infusion, Follow-up

Content	Screen	Leuk-apheresis	Before FC	FC	Before CAR-T infusion	CAR-T infusion	Follow-up 1	Follow-up 2	Follow-up 3	Follow-up 4	Follow-up 5	Follow-up 6	Follow-up 7	Follow-up 8	Follow-up 9	Follow-up 10	Long term follow-up
Weeks/ Months	-3	-3~-2		-1		1	1	1	1	2	2	14w/3 m	24w/6 m	36w/9 m	48w/1 y	1y-2y	2y-3 y
Days	-28	-21~-14		-5 -3		0	1	4	7	10	14	Every 3 m	Every 3 m	Every 3 m	Every 3 m	Every 6 m	Annually
ECOG score	X						X	X	X	X	X	X	X	X	X	X	X
Eligibility	X																
**Tumor and disease assessment**																	
History of treatment	X																
Tissue biopsy[1]	X																
Bone marrow examination [2]	X																
Immune typing	X																
CT ultrasound [3]	X																

PET-CT evaluation [4]	X											X	X	X	X	X	X
Demographic study [5]	X																
Medical history	X																
body surface [6]	X	X	X		X												
Physical examination [7]	X		X		X		X	X	X	X	X	X	X	X	X	X	X
Mini-Mental State Examination (MMSE)	X	X	X	X	X	X	X	X	X	X	X	X	X	X	X	X	X
**Treatment process**																	
Inform Consent Form	X																
Leuk-apheresis		X															
FC Chemo-therapy				X													
CAR-T infusion						X											

Electro-cardiograph monitoring [8]						X											
Vital signs [9]	X			X		X	X	X	X	X	X	X	X	X	X	X	
Oxygen saturation	X			X		X	X	X	X	X							
Adverse event [10]	X	X		X		X	X	X	X	X	X	X	X	X	X	X	X
**Clinical laboratory examination**																	
Blood routine test/CRP	X		X		X		X	X	X	X	X	X	X	X	X	X	X
Blood biochemistry	X		X		X		X	X	X	X	X	X	X	X	X	X	X
Cytokines	X		X		X		X	X	X	X	X	X	X	X	X	X	X
Infectious diseases/ Viruses [11]	X																
Urine pregnancy test	X		X		X												
Ferritin			X		X		X	X	X	X	X	X	X	X	X	X	X
LDH			X		X		X	X	X	X	X	X	X	X	X	X	X
Immune globulin			X		X		X	X	X	X	X	X	X	X	X	X	X

**Central laboratory examination**																	
CAR gene copies in peripheral blood			X		X		X	X	X	X	X	X	X	X	X	X	X
Proportion of CAR+ T cells in peripheral blood			X		X		X	X	X	X	X	X	X	X	X	X	X
Peripheral blood lymphocyte subsets[12]			X		X		X	X	X	X	X	X	X	X	X	X	X

Footnote

1. The biopsy sequence contained CD19 expression and was acceptable for case diagnosis reports within the first 6 months of enrollment. Patients assessed for disease progression or relapse after remission following CAR-T therapy should be biopsied again and case reports provided, with no more than two biopsies per patient.

2. Bone marrow cytology involves bone marrow aspiration or biopsy. DLBCL subjects may have a PET-CT instead of a bone marrow test. If the PET-CT indicates a bone marrow negative, the investigator will decide whether to have a bone marrow test.

3. The examination results within 4 weeks prior to signing the informed consent form can be used as data for screening. For imaging evaluation, enhanced CT was used to identify lymphoma lesions, such as chest CT plain scan for patients allergic to CT contrast agent, and enhanced MRI was available for skull,

abdominal cavity, abdominal cavity, and pelvic cavity. Subjects should use the same imaging technique throughout the study period.

4．The time points of PET-CT examination were screening period, 3 months, 6 months, 9 months, 12 months, and every 6 months after one year.

5. Demographic information, including date of birth, sex, ethnicity, etc.

6. Body surface area is calculated.

7. The physical examination includes general conditions, skin, head, eyes, ears, nose, throat, heart, lungs, chest, abdomen, limbs, nervous system, back/spinal cord, and lymph nodes.

8. Routine Electrocardiogram (ECG) monitoring was performed from 30 minutes before CAR-T cell infusion to 2 h after infusion (every 30 minutes from 30 minutes before infusion to 1 h after infusion; After 1 h, record once every 1 h). If the vital signs were unstable after CAR-T cell-infusion, the monitoring time should be extended until the vital signs were stable.

10. AE and serious adverse events will be collected and monitored closely for 3 years between the patient's signing of the informed consent and treatment. Possible or certain study-related serious adverse events, including secondary tumors, were collected after cell infusion.

11. Infectious disease and virus surveillance, including HBC set, HBV DNA (only when HbcAg positive), HCV-Ab, human immunodeficiency virus (HIV) antibodies, TPPA (Treponema pallidum specific antibody).

12. Lymphocyte subtypes, including $CD19^{+}$, $CD20^{+}$ B lymphocytes, $CD3^{+}$T lymphocytes, CD4/$CD8^{+}$T cells ratio, etc.

2.7 Study Withdrawal

It is expected that the most common reason for withdrawal from the study will be disease progression. Subjects, however, may withdraw from this study at any time, for any reason. Other than progressive disease or death, other possible reasons for study withdrawal include:

- Toxicity
- Subject preference, including decision to undergo alternative treatment
- Physician preference
- Failure of CAR-T production
- Closure of the study

It is strongly requested that subjects who respond, but then develop progressive disease, undergo a tumor biopsy prior to study discontinuation.

2.8 Suspension Criteria

Subjects who did not complete the protocol were considered to stop the trial as soon as possible. Cause of premature termination such as voluntary evacuation, toxic side effects and death, must be documented in case reports. The research evaluation will be completed at the end of the suspension.

Follows are the potential reasons for premature suspension:

(1) The follow-up of this subject failed;

(2) The principal investigator believe that the disease is too serious to continue;

(3) Patients do not comply with the treatment and clinical agreement of the study;

(4) Voluntary withdrawal;

(5) Failure to producing clinical cell doses that meet the quality control standards.

2.9 Study Population

This study will enroll R/R LBCL patients who met enrollment criteria.

2.10 Number of Subjects

Part A (Dose Escalation phase): Initially, up to approximately 9-18 evaluable subjects will be enrolled. Depending on the outcome of these cohorts and decisions made by the investigator.

Part B (Expansion Phase):

Hypothesis

The planned dose used in the expansion phase is based on the review of the preliminary safety generated in Part A of the study and further, differentiates between a treatment that has a true response rate of 25% or less and a treatment with a true response rate of 55% or more. The hypothesis is that the objective response rate to 7 × 19 CAR-T in the R/R LBCL is significantly greater than 30%.

Sample Size considerations

Part B uses a single-arm design to test for an improvement in response rate in the R/R LBCL. For the test of efficacy, this study has ≥ 90% power to distinguish between an active therapy with a 55% ORR from therapy with an ORR of 25% or less with a 1-sided alpha of 0.025, considering a 10% dropout rate, a total of 27 samples is required.

Statistical Assumptions

Treatment outcomes for R/R LBCL are provided in Table 2-4 below. To evaluate the validity of the assumption on the underlying response rate, retrospective studies (historical data and database reviews) of the response rate in the target population will be conducted. As shown, the patient response to conventional treatments (including radiotherapy, and chemotherapy) varies between 14% and 23%. Meanwhile, the response to CAR-T cell therapy lies within the range of 52% to 73%. Based on these data, this study assumes that the underlying objective response rate (in the conventional treatments) among the relapsed or refractory population is estimated to be 25% and that an improvement in the response rate to 55% provides clinically meaningful benefit.

Table 2-4 Historical Responses in R/R LBCL

Setting	Outcome to Subsequent Therapy
**Conventional**	
Ardeshna et al 2005 (n=28)[2]	ORR 18%
Seshadri et al 2008 (n=73) [3]	ORR 14%
Telio et al 2012 (n=111)[4]	ORR 23%
Phillip et al 1995 (n=28)[5]	ORR 21%
Crump et al 2014 (n= 189)[6]	ORR 26%
**CAR-T**	
Abramson et al 2020 (n=344)[7]	ORR 73%
Schuster et al 2018 (n=93)[8]	ORR 52%
Xuan Zhou et al 2020 (n=21)[9]	ORR 67%

Ying Z et al 2021(n=58)[10]	ORR 60.3%

2.11 Study Eligibility

Inclusion Criteria:

(1) Male or female participants aged ≥ 18 and ≤ 75 years at time of enrollment;

(2) Eastern Cooperative Oncology Group (ECOG) performance status less than or equal to 3 (Table 2-5);

(3) Histologically confirmed CD19$^+$ B-cell lymphoma according to the World Health Organization 2008 criteria, including

Diffuse large B-cell lymphoma (DLBCL);

Primary mediastinal B-cell lymphoma (PMBCL);

Transformed follicular lymphoma (tFL);

Mantle cell lymphoma (MCL) ;

Refractory DLBCL, PMBCL and tFL were defined according to the SCHOLAR-1 study[11]: progressive disease (received ≥4 cycles of first-line therapy) or stable disease (received 2 cycles of later-line therapy) lasting ≤ 6 months as best response to chemotherapy regimen that should included rituximab and anthracycline; or relapse ≤ 12 months after prior auto-hematopoietic stem-cell transplantation (HSCT);

Refractory MCL was defined as refractory/relaspe after prior above-mentioned immunochemotherapy and ibrutinib, or not agree to receive ibrutinib;

(4) Patients had failure with, be ineligible for, or not consent to HSCT;

(5) At least has one measurable tumor, the length diameter of any lymph node should be greater than 1.5 cm or the length diameter of any external node should be greater than 1.0 cm, and the lesion should be taken in by PET-CT (SUV is larger than the liver blood pool);

(6) Life expectancy ≥12 weeks;

(7) Included the following laboratory values:

- An absolute neutrophil count > 1000 per μL, platelets count ≥ 45,000 per μL, and hemoglobin > 8.0 g dL-1;
- Alanine aminotransferase and aspartate aminotransferase ≤ 2.5 × the upper limit of the normal range (ULN), and total bilirubin ≤ 2.0 mg dL-1;
- A serum creatinine of ≤ 1.5 × ULN;
- Left ventricular ejection fraction ≥50%;

(8) Informed Consent/Assent: All subjects have the ability to understand and the willingness to sign a written informed consent.

Exclusion Criteria:

(1) History of other malignancies;

(2) Auto-HSCT within 6 weeks of informed consent;

(3) Prior chimeric antigen receptor T cell therapy or other genetically modified T cell therapy with 3 months;

(4) Chemotherapy other than lymphodepleting chemotherapy, therapeutic doses of steroids, immunosuppressive agent, lenalidomide, bortezomib, ibrutinib, or any radiation therapy within 2 weeks before leukapheresis;

(5) Investigatory drug within 4 weeks before screening;

(6) Primary immunodeficiency and HIV infection;

(7) Clinically significant active infection (simple urinary infection, bacterial pharyngitis allowed) or currently received IV antibiotics or have received IV antibiotics within 7 days prior to enrollment;

(8) Active hepatitis B or hepatitis C. Hepatitis B: HBV-DNA ≥ 1,000 IU/mL; Hepatitis;

(9) Subjects with detectable cerebrospinal fluid malignant cells, or brain metastases, or with a history of cerebrospinal fluid malignant cells or brain metastases;

(10) Currently pregnant or refusal to practice birth control within 1 years.

(11) creatinine clearance <30 mL/min.

Table 2-5. Performance Status Based on the Eastern Cooperative Oncology Group (ECOG) Scale

The ECOG score runs from 0 to 5 score, with 0 denoting perfect health and 5 death:

0	Asymptomatic (Fully active, able to carry on all pre-disease activities without restriction)
1	Symptomatic but completely ambulatory (Restricted in physically strenuous activity but ambulatory and able to carry out work of a light or sedentary nature. For example, light housework, office work)
2	Symptomatic, < 50% in bed during the day (Ambulatory and capable of all self-care but unable to carry out any work activities. Up and about more than 50% of waking hours)
3	Symptomatic, > 50% in bed, but not bed-bound (Capable of only limited self-care, confined to bed or chair 50% or more of waking hours)
4	Bed-bound (Completely disabled. Cannot carry on any self-care. Totally confined to

	bed or chair)
5	Death

2.12 Exploratory

- Biomarker studies including Cytokines, serum C-reactive protein (CRP) levels and CAR gene copies in peripheral blood
- Endpoints related to product characterization (T-cell phenotype in the CAR-T products, duration of cell manufacturing time, transduction ratios).

2.13 Covariates

- ECOG performance status at baseline (0-1 vs 2-3)
- Age at baseline
- Sex (male, female)
- Disease type (DLBCL, PMLBCL, MCL, and tFL)
- Refractory subgroup (refractory vs relapsed)
- Subgroup (germinal center B cell-like (GBC), non-GCB)
- International prognostics index (IPI) risk category at screening score (0-1, 2-3, and 4-5)
- Extranodal sites (≤1 vs >1)
- Lactate dehydrogenase (LDH) (normal vs elevated)
- CRS grade (0-1 vs 3-4)
- Tocilizumab (Used vs Not used)
- Glucocorticoid (Used vs Not used)

- Disease stage (I, II, III, IV) and extent (presence of B symptoms, bulky disease, extranodal disease) at as determined by the investigator at screening
- Number of prior chemotherapy regimens

2.14 Clinical efficacy evaluation

Response was determined by whole-body PET-CT at month 3 according to the Lugano 2014 classification (2014)[1].

Objective Response Rate (ORR) means the proportion of subjects with either a CR or PR evaluated at three months after CAR-T cell infusion. All subjects who do not meet the criteria for objective response by the analysis data cutoff date will be considered non-responders.

Duration of response (DOR) is defined only for subjects who experience an objective response and is the time from the first objective response to disease progression or death due to disease relapse.

Progression-free Survival (PFS) is defined as the time from the CAR-T infusion date to the date of disease progression or death from any cause.

Overall Survival (OS) is defined as the time from CAR-T infusion to the date of death from any cause.

The PET/CT is used to evaluate the response every 3 months after CAR-T infusion compared with baseline disease status before treatment, and the objective response was classified into four parts of CR, PR, SD or PD at each time point. ORR, OS, PFS, and DOR were used to assess the clinical efficacy.

2.15 Adverse Events

Adverse events for 7 × 19 CAR-T treatment include CRS, Neurological toxicity, hematological toxicity, infections, auto-immune disorders, and secondary malignancies. The classification of CRS and Neurological toxicity was according to the American Society for Transplantation and Cellular Therapy (ASTCT, also known as Lee, 2019)[12]. Other adverse events were graded to the guide of CTCAE 4.03.

All adverse events occurring during the adverse event reporting period must be recorded. Adverse events (including lab abnormalities that constitute AEs) should be described using a diagnosis whenever possible, rather than individual underlying signs and symptoms. When a precise diagnosis cannot be identified, each sign or symptom should be reported as a separate Adverse Event. All adverse events should be treated appropriately as far as possible; each adverse event should be evaluated to determine:

(1) The severity grade (CTCAE4.03/ ASTCT);

(2) Its duration (start and end dates);

(3) Its relationship to the study treatment: is there a reasonable possibility that the AE is related to the study treatment-No (unrelated) or Yes. If yes, is the event possible, probably, or definitely related to the investigatory treatment or the non-investigatory treatment (i.e. lymphodepleting chemotherapy);

(4) Action taken with respect to study or investigatory treatment (none, dose adjusted, temporarily interrupted, permanently discontinued, unknown, not applicable);

(5) Whether medication or therapy taken (i.e., no concomitant medication/non-drug therapy, concomitant medication/non-drug therapy).

Adverse events of interest for CAR-T treatment include adverse events in the categories of:

Identified risks:

• Neurological toxicity

•CRS

• Cytopenias (including febrile neutropenia)

• B-cell aplasia

Potential risks:

• Infections

• Auto-immune disorders

• Secondary malignancies

• Tumor lysis syndrome

Severe Adverse Events (SAEs)

SAEs are defined as events that require hospitalization, need to prolonged hospitalization time, impair work ability, and endanger life or result in death during this trial. Any SAEs that occur during the treatment or observation phase, whether or not it is related to the CAR T therapy, must be reported to the coordinating organization/its representative within 24 h of his/her discovery.

CRS Definition and Management

The primary acute toxicity observed to date with B cell malignancy-targeted CAR-T cells are CRS, and this protocol will follow the recommendations and management for CRS as defined by ASTCT, which listed in Table 2-6, 2-7, and 2-8, respectively. For this protocol, a CRS is defined as a constellation of symptoms that may include (but are not limited to) fever, chills, hypotension, dyspnea, hypoxia, confusion, mental status changes, seizures, myalgias, nausea and vomiting, and laboratory abnormalities including elevated AST, ALT, bilirubin, D-dimers, ferritin, urea and/or creatinine. Cytokine panels are also typically markedly abnormal but are considered exploratory in nature. The work-up of CRS should include hospitalization and evaluation for an infection etiology (e.g., blood cultures, urine culture, chest X-ray, as required). In addition, any subject hospitalized for fever or work-up of CRS should have blood drawn for CAR^+ T cells and cytokine assessments along with study-outlined chemistries and hematology including ferritin, fibrinogen, lactate dehydrogenase (LDH), and C-reactive Protein.

Table 2-6. ASTCT CRS Consensus Grading

CRS Parameter	Grade1	Grade2	Grade3	Grade4
Fever*	Temperature ≥38 °C	Temperature ≥38 °C	Temperature ≥38 °C	Temperature ≥38 °C
		With		
Hypotension	None	Not requiring vasopressors	Requiring a vasopressor with or without vasopressin	Requiring multiple vasopressors (excluding vasopressin)
		And/Or#		
Hypoxia	None	Requiring low-flow nasal cannula$ or blow-by	Requiring high-flow nasal cannula, facemask, nonrebreather mask, or Venturi mask	Requiring positive pressure (eg.CPAP, BiPAP, intubation and mechanical ventilation)

Organ toxicities associated with CRS may be graded according to CTCAE v5.0 but they do not influence CRS grading.

* Fever is defined as temperature ≥38 °C not attributable to any other cause. In patients who have CRS then receive antipyretic or anticytokine therapy such as tocilizumab or steroids, fever is no longer required to grade subsequent CRS severity. In this case, CRS grading is driven by hypotension and/or hypoxia.

CRS grade is determined by the more severe event: hypotension or hypoxia not attributable to any other cause. For example, a patient with temperature of 39.5 °C, hypotension requiring 1 vasopressor, and hypoxia requiring low-flow nasal cannula is classified as grade 3 CRS.

$ Low-flow nasal cannula is defined as oxygen delivered at ≤6 L/minute. Low flow also includes blow-by oxygen delivery, sometimes used in pediatrics. High-flow nasal cannula is defined as oxygen delivered at >6 L/minute.

Table 2-7. Clinical Signs and Symptoms Associated with CRS

Organ System	Symptoms
**Constitutional**	Fever+/-rigors, malaise fatigue, anorexia, myalgias, arthralgias, nausea, vomiting, headache
**Skin**	Rash
**Gastrointestinal**	Nausea, vomiting, diarrhea
**Respiratory**	Tachypnea, hypoxemia
**Cardiovascular**	Tachycardia, widened pulase pressure, hypotension, increase cardiac output (early), potentially diminished cardiac output (late)
**Coagulation**	Elevated D-Dimer, hypofibrinogenemia+/-bleeding
**Renal**	Azotemia
**Hepatic**	Transaminitis, hyperbilirubinemia
**Neurologic**	Headache, mental status changes, confusion, delirium, word finding difficulty or frank aphasia, hallucinations, tremor, dysmetria, altered gait, seizures

Table 2-8. Treatment guidelines for CRS

Grading assessment of CRS	Widespread comorbidities or older age?<br>No/Yes	Treatment
Grade1<br>• Fever (defined as ≥ 38.3 °C)<br>• Systemic symptoms	N/A	• Vigilant supportive care<br>• Assess infection<br>• If fever and neutropenia develop, treat them, monitor fluid balance, and use antipyretics and analgesics<br>• as needed
Grade 2<br>• Hypotension: Responds to fluids or a low dose of vasopressin<br>• Hypoxia: Response < 40% O^2<br>• Organotoxicity: Grade 2	No	• As described in Grade 1<br>• Closely monitor cardiac and other organ function
Grade 2<br>• Hypotension: Responds to fluids or a low dose of vasopressin<br>• Hypoxia: Response < 40% O^2	Yes	• As described in Grade 2<br>• Consider tolumab (8 mg/kg) ± corticosteroid (e.g. Methylprednisolone 1 mg/kg BID) or dexamethasone 10 mg q6hrs

Grade 3<br>• Low blood pressure: Multiple vasopressin or high doses of vasopressin [a] are required<br>• Oxygen deficiency: ≥40% O2 is required<br>• Organotoxicity: Grade 3 or 4 elevated aminotransferase	N/A	
Grade 4<br>• Mechanical ventilation<br>• Organotoxicity: Grade 4 does not include elevated transaminase	N/A	• As described in Grade 2/3<br>• Corticosteroids (ex: methylprednisolone 1g/d × 3 followed by rapid decline consisting of 250 mg BID × 2 days, 125 mg, BID × 2 days, then 60 mg BID ×2 days)

a is seen in Table 2-9

Table 2-9. High Dose Vasopressors (all doses are required for ≥ 3 hs)

Pressor	Dose
**Norepinephrine monotherapy**	≥ 20 μg/min
**Dopamine monotherapy**	≥ 10 μg/kg/min
**Phenylephrine monotherapy**	≥ 200 μg/min
**Epinephrine monotherapy**	≥ 10 μg/min
**If on vasopressin**	Vasopressin + norepinephrine is equivalent to ≥ 10μg/min
**If on combination vasopressors (not vasopressin）**	Noradrenaline is equivalent to ≥ 20 μg/min*

*VASST Trail vasopressor equivalent equation: norepinephrine equivalent dose=[norepinephrine (μg/min)]+ [dopamine (μg/min)/2]+ [epinephrine (μg/min)]+ [phenylephrine (μg/min)/10]

Immune effector cell-associated neurotoxicity syndrome (ICANS)

ICANS have been reported in anti-CD19 CAR-T cell studies, including confusion, obtundation, aphasia, and myoclonus, the reported neurologic toxicities in these anti-CD19 CAR-T cells studies have been often been transient; however, there is evident that persistent neurologic toxicity may also occur. The mechanism of action of these neurologic toxicities remains unclear and under investigation. Because these syndromes are only now being characterized in the setting of CAR T cell therapy, in the event of neurologic toxicity it is recommended investigators thoroughly assess subjects including the use of MRI and lumbar punctures to investigate possible mechanisms of action. The Grading and management for Neurologic Toxicity were followed by the guidelines listed in Table 2-10, and Table 2-12, respectively.

Table 2-10. ASTCT ICANS Consensus Grading

Neurotoxicity Domain	Grade 1	Grade 2	Grade 3	Grade 4
**ICE score***	7-9	3-6	0-2	0 (patient is unarousable and unable to perform ICE)
**Depressed level Of consciousness#**	Awakens spontaneously	Awakens to voice	Awakens only to tactile stimulus	Patient is unarousable or requires vigorous or repetitive tactile stimuli to arouse. Stupor or coma
**Seizure**	N/A	N/A	Any clinical seizure focal or generalized that resolves rapidly or nonconvulsive seizures on EEG that resolve with intervention	Life-threatening prolonged seizure (>5 min); or Repetitive clinical or electrical seizures without return to baseline in between
**Motor findings$**	N/A	N/A	N/A	Deep focal motor weakness such as hemiparesis or paraparesis
**Elevated ICP/ cerebral edema**	N/A	N/A	Focal/local edema on neuroimagingx	Diffuse cerebral edema on neuroimaging; decerebrate or decorticate posturing; or cranial nerve VI palsy; or papilledema; or Cushing's triad

ICANS grade is determined by the most severe event (ICE score, level of consciousness, seizure, motor findings, raised ICP/cerebral edema) not attributable to any other cause; for example, a patient with an ICE score of 3 who has a generalized seizure is classified as grade 3 ICANS.ICE score see Table 2-11.

N/A indicates not applicable.

* A patient with an ICE score of 0 may be classified as grade 3 ICANS if awake with global aphasia, but a patient with an ICE score of 0 may be classified as grade 4

ICANS if unarousable.

Depressed level of consciousness should be attributable to no other cause (eg, no sedating medication).

$ Tremors and myoclonus associated with immune effector cell therapies may be graded according to CTCAE v5.0, but they do not influence ICANS grading.

Intracranial hemorrhage with or without associated edema is not considered a neurotoxicity feature and is excluded from ICANS grading. It may be graded according to CTCAE v5.0.

Table 2-11. ICE Score

**Parameter**	**Score**
Orientation: year, month, city, hospital	4
Naming: ability to name 3 objects (eg, point to clock, pen, button)	3
Following commands: ability to follow simple commands<br><br>(eg, “show me 2 fingers” or “close your eyes and stick out your tongue”)	1
Writing: ability to write a standard sentence (eg, “our national bird is the bald eagle”)	1
Attention: ability to count backwards from 100 by 10	1

Scoring:

10, no impairment

7-9, grade 1 ICANS

3-6, grade 2 ICANS

0-2, grade 3 ICANS

0 due to patient unarousable and unable to perform ICE assessment, grade 4 ICANS

Table 2-12. Treatment guidelines for ICANS

Neurotoxicity	Treatment	Evaluation
Grade 1: Examples include:<br>(1) lethargy - mild drowsiness or sleepiness;<br>(2) Blurred consciousness-mild disorientation;<br>(3) Encephalopathy - mildly restrictive ADL;<br>(4) dysplasia - does not affect communication ability;<br>(5) simple partial seizure; Unconscious loss	Vigilant supportive care	Neurological examination<br><br>Perform additional tests based on clinical indications
Grade 2: Examples include:<br>(1) Severe drowsiness and limited instrumental ADL;<br>(2) Confusion and moderate disorientation; Restricted instrument ADL;<br>(3) Encephalopathy - instrumental ADL limitation;<br>(4) Dysplasia-moderate influence on active communication ability; Transient generalized epilepsy.	(1) Vigilant supportive care;<br>(2) Consider prophylactic antiepileptic medication (e.g., levetiracetam 500 mg, BID);<br>(3) If comorbiditis exists (e.g., grade 2 or higher CRS), consider using 8 mg/kg	

	tocilizumab within 1 h (no more than 800 mg).	Brain MRI and cerebrospinal fluid evaluation should be included in addition to neurological examination.<br><br>Ecg is considered as clinical indication.
Grade 3: Examples include:<br>(1) letharg-lethargy or stupor;<br>(2) Blurred consciousness-severe disorientation; Restricted self-care ADL;<br>(3) Limited ADL of encephalopathy-life autorationality;<br>(4) Dysplasia-severe receptivity or emotive traits, affecting the ability to read, write, or communicate clearly, but still having multiple seizures despite drug intervention;<br>(5) Fatigue, limited self-care ADL; Incapacitated;<br>Complete stool/bladder incontinence;	(1) Consider giving tolizumab 8 mg/kg IV (not more than 800 mg) within 1 h;<br>(2) If symptoms are unstable or do not improve, tolizumab should be repeated every 4-6 h;<br>(3) Consider corticosteroids (e.g. Dexamethasone 10 mg IV every 6 h, methylprednisolone 1 mg/kg, BID) if symptoms worsen despite tocilizumab administration;<br>(4) Consider the use of prophylactic antiepileptic drugs.	

Grade 4: Examples include:<br>(1) The consequences are life-threatening;<br>(2) Emergency measures are required;<br>(3) mechanical ventilation;<br>(4) life-threatening, long-term repeated seizures;	Corticosteroids (ex: methylprednisolone 1 g/d × 3 days followed by a rapid decline consisting of 250 mg, BID × 2 days, 125 mg, BID × 2 days, then 60 mg, BID × 2 days).	

2.16 Laboratory Test

Clinical Laboratory Tests

Clinical laboratory tests (Table 2-13) include Hematology test, Serum Chemistry, Coagulation function test and Enzymes & Liver functions.

Table 2-13. Clinical Laboratory Tests

Hematology	Serum Chemistry	Coagulation	Enzymes & Liver Functions
CBC with differential	Sodium	Prothrombin time	AST
Ferrintin	Potassium, chloride	(PT)/partial	ALT
Fibrinogen	Bicarbonate	thromboplastin time	Alkaline phosphatase
	Glucose	(PTT), international	Total and direct
	Uric acid	normalized ratio	bilirubin
	Phosphate	(INR)	Albumin
	Magnesium		LDH
	C-reactive protein		

Abbrev: CBC, complete blood count; AST, aspartate aminotransferase; ALT, alanine aminotransferase; LDH, lactate dehydrogenase

CBC includes hematocrit, hemoglobin, red blood cell (RBC) count, white blood cell (WBC) count with differential, and platelet count.

Additional Eligibility-Determining Laboratory Tests

During Screening, blood samples will be collected for additional eligibility-determining laboratory tests, as follows:

Screening serology will be evaluated using standard methods. The serology panel should include the following:

- Human immunodeficiency virus (HIV)
- Hepatitis B virus core antibody (HBcAb)
- Hepatitis B virus surface antibody (HBsAb)
- Hepatitis B virus surface antigen (HBsAg)
- Hepatitis C virus (HCV) antibody
- HCV RNA
- Syphilis
- Cytomegalovirus Antibody

Blood may also be drawn for additional serology testing if subject has risk factors or clinical evidence of infection with other communicable disease agents or disease.

Serology should be performed within 7 days prior to leukapheresis. Additional serology may be performed if required according to country-specific and institutional guidelines.

CAR^+ T cells measured in peripheral blood

The level of CAR^+ T in peripheral blood post-infusion was assessed by flow cytometry and PCR assay at day 1, day 4, day 7, day 11 and week 2, month 1, month 3, month 6, month 12, month 18, month 24, and month 36. The maximum CAR^+ T cell level and the time at which the maximum level was attained.

Lymphocyte subsets

The levels of lymphocytes and the subject incidence of lymphopenia, B-cell aplasia, and the subject incidence of recovery after lymphopenia and B cell aplasia will be collected for each subject based on lymphocyte subsets measured before conditioning chemotherapy, on the day of CAR-T infusion, week 4,

month 3, month 6, month 9, month 12, month 15, month 18, month 24 and month 36. Among subjects who experience lymphopenia or B-cell aplasia, summary statistics for the time to the onset of these conditions will be provided. The duration of lymphopenia and B-cell aplasia will be summarized; the duration of these events for subjects with persistent lymphopenia or B-cell aplasia at the last lymphocyte measurement will be censored at that time.

Detection of Serum Cytokines

Cytokine analysis will be performed in serum before and after CAR-T infusion. Examples of inflammatory cytokines that should be measured include IL-2, IL-4, IL-10, IL-6, TNF-α and IFN-γ.

2.17 Statistical Procedures

Sample Size Estimation

The sample size in Phase 1 trial is determined by the role of 3 + 3 dose escalation.

Based on the planned expansion phase study up to approximately 27 evaluable subjects will be enrolled in expansion phase study (part B) of the study.

Populations for Analysis

The following subject population will be evaluated:

Intent-to-treat (ITT) population: All subjects who receive 7 × 19 CAR-T cells are included for the analysis of safety and efficacy.

The MTD population: The subjects in escalation phase are included for determining the MTD.

Efficacy population: All subjects in Part A and Part B of the study are included for analysis of efficacy.

Interim Analysis

There will be no formal interim analysis of the data. Interim safety reviews will be performed by the Safety Review Committee (SRC) following completion of every dosing cohort in the dose escalation phase prior to next dose escalating. The SRC will also evaluate all data of safety prior to the escalation phase (Part B study).

Statistical Methods

General methods: Statistical analyses will be primarily descriptive in nature. Correlative tabulations will be produced from the data of all subjects both Part A and Part B study.

Demographic, baseline characteristics and disposition of subjects: Demographic, baseline characteristics and disposition of patients treated with CAR-T, such as disease, prior treatment, and ECOG PS will be summarized. Baseline values of all efficacy parameters should be included in tables of baseline, post-baseline and and change from baseline, with all subjects data included in the summary statistics included in by-subject, by-time data listings.

Efficacy and Pharmacokinetic Analyses: The proportion of subjects who meet response criteria after CAR-T treatment will be tabulated along with a 90% exact binomila confidence interval.

The probabilities of OS and PFS are estimated using Kaplan-Meier method, and survival curves will be compared between groups with a log-rank test. PFS and OS are defined as the time from CAR-T cells infusion to first relapse or death, with censoring at the last follow-up. DOR is defined only for subjects with a response at 3 months and is the time from the first responses to disease progression or death, with censoring at the last follow-up.

Reference

1 Cheson, B. D. *et al.* Recommendations for initial evaluation, staging, and response assessment of Hodgkin and non-Hodgkin lymphoma: the Lugano classification. *J Clin Oncol* **32**, 3059-3068 (2014).

2 Ardeshna, K. M. *et al.* Conventional second-line salvage chemotherapy regimens are not warranted in patients with malignant lymphomas who have progressive disease after first-line salvage therapy regimens (2005).

3 Seshadri, T., Kuruvilla, J., Crump, M. & Keating, A. Salvage therapy for relapsed/refractory diffuse large B cell lymphoma. *Biol Blood Marrow Transplant* **14**, 259-267 (2008).

4 Telio, D. *et al.* Salvage chemotherapy and autologous stem cell transplant in primary refractory diffuse large B-cell lymphoma: outcomes and prognostic factors. *Leuk Lymphoma* **53**, 836-841 (2012).

5 Philip, T. *et al.* Autologous bone marrow transplantation as compared with salvage chemotherapy in relapses of chemotherapy-sensitive non-Hodgkin's lymphoma. *N Engl J Med* **333**, 1540-1545 (1995).

6 Crump, M. *et al.* Randomized comparison of gemcitabine, dexamethasone, and cisplatin versus dexamethasone, cytarabine, and cisplatin chemotherapy before autologous stem-cell transplantation for relapsed and refractory aggressive lymphomas: NCIC-CTG LY.12. *J Clin Oncol* **32**, 3490-3496 (2014).

7 Abramson, J. S. *et al.* Lisocabtagene maraleucel for patients with relapsed or refractory large B-cell lymphomas (TRANSCEND NHL 001): a multicentre seamless design study. *Lancet* **396**, 839-852 (2020).

8 Schuster, S. J. *et al.* Tisagenlecleucel in Adult Relapsed or Refractory Diffuse Large B-Cell Lymphoma. *N Engl J Med* **380**, 45-56 (2019).

9 Zhou, X. *et al.* Phase I Trial of Fourth-Generation Anti-CD19 Chimeric Antigen Receptor T Cells Against Relapsed or Refractory B Cell Non-Hodgkin Lymphomas. *Front Immunol* **11**, 564099 (2020).

10 Ying, Z. *et al.* Relmacabtagene autoleucel (relma-cel) CD19 CAR-T therapy for adults with heavily pretreated relapsed/refractory large B-cell lymphoma in China. *Cancer Med* **10**, 999-1011 (2021).

11 Crump, M. *et al.* Outcomes in refractory diffuse large B-cell lymphoma: results from the international SCHOLAR-1 study. *Blood* **130**, 1800-1808 (2017).

12 Lee, D. W. *et al.* ASTCT Consensus Grading for Cytokine Release Syndrome and Neurologic Toxicity Associated with Immune Effector Cells. *Biol Blood Marrow Transplant* **25**, 625-638 (2019).
